# A novel multi-hybrid differential evolution algorithm for optimization of frame structures

**DOI:** 10.1038/s41598-024-54384-3

**Published:** 2024-02-28

**Authors:** Rohit Salgotra, Amir H. Gandomi

**Affiliations:** 1 Faculty of Physics and Applied Computer Science, AGH University of Science and Technology, Kraków, Poland; 2 MEU Research Unit, Middle East University, Amman, Jordan; 3 Faculty of Engineering and IT, University of Technology Sydney, Ultimo, NSW 2007 Australia; 4 University Research and Innovation Center (EKIK), Óbuda University, 1034 Budapest, Hungary

**Keywords:** Differential evolution, Hybridization, Self-adaptive parameters, Numerical optimization, Frame structure design, Swarm intelligence, Engineering, Mathematics and computing

## Abstract

Differential evolution (DE) is a robust optimizer designed for solving complex domain research problems in the computational intelligence community. In the present work, a multi-hybrid DE (MHDE) is proposed for improving the overall working capability of the algorithm without compromising the solution quality. Adaptive parameters, enhanced mutation, enhanced crossover, reducing population, iterative division and Gaussian random sampling are some of the major characteristics of the proposed MHDE algorithm. Firstly, an iterative division for improved exploration and exploitation is used, then an adaptive proportional population size reduction mechanism is followed for reducing the computational complexity. It also incorporated Weibull distribution and Gaussian random sampling to mitigate premature convergence. The proposed framework is validated by using IEEE CEC benchmark suites (CEC 2005, CEC 2014 and CEC 2017). The algorithm is applied to four engineering design problems and for the weight minimization of three frame design problems. Experimental results are analysed and compared with recent hybrid algorithms such as laplacian biogeography based optimization, adaptive differential evolution with archive (JADE), success history based DE, self adaptive DE, LSHADE, MVMO, fractional-order calculus-based flower pollination algorithm, sine cosine crow search algorithm and others. Statistically, the Friedman and Wilcoxon rank sum tests prove that the proposed algorithm fares better than others.

## Introduction

The field of optimization research has experienced significant growth in recent decades, particularly with the widespread utilization of nature-inspired optimization algorithms (NIAs). These algorithms, derived from natural phenomena, are now employed across a multitude of research domains, including engineering design, management science, medical technology, social science, and others. While genetic algorithms (GA)^1^, differential evolution (DE)^2^, and particle swarm optimization (PSO)^3^ remain influential, the landscape has expanded with the introduction of numerous new algorithms inspired by different species and natural processes. This continuous innovation and the development of hybrid techniques underscore the dynamic nature of NIAs, showcasing their relevance and applicability in diverse problem-solving scenarios.

The fundamental domain is categorized into two main classes: evolutionary algorithms (EAs) and swarm intelligent algorithms (SIAs). EAs are rooted in fundamental natural processes, specifically drawing inspiration from Darwinian theory and natural selection. Notable examples include GA^1^, memetic algorithm (MA)^4^, scatter search (SS)^5^, stochastic fractal search (SFS)^6^, fire-hawk algorithm (FHA)^7^ among others, as prominent representatives.

On the other hand, SIAs are built upon the collective behavior observed in various species. This category encompasses algorithms such as red fox optimization algorithm (RFO)^8^, mud ring algorithm (MRA)^9^, sea horse optimizer (SHO)^10^, escaping bird search (EBS)^11^, golden eagle optimizer (GEO)^12^, clouded leopard optimization (CLO)^13^, hermit crab shell exchange (HCSE)^14^, honey badger algorithm (HBA)^15^, naked mole rat algorithm^16^ cuckoo search algorithm (CS)^17^, whale optimization algorithm (WOA)^18^, grey wolf optimization (GWO)^19,20^, equilibrium optimizer (EO)^21^, moth flame optimization (MFO)^22^ and others. These algorithms leverage the swarming behaviour of different species as a basis for their optimization strategies.

In real world, most of the practical engineering design problems are highly challenging and differential evolution (DE) has been applauded as an efficient problem solver by the evolutionary computing community, due to its simple linear structure, lesser known tuning parameters and versatile applicability^23^. The major reason for its popularity is because of its splendid performance and ranking in Congress on Evolutionary Computation (CEC) competitions by IEEE for various complex domain research scenarios and benchmark test suites (such as multi-modal, composite, single objective, dynamic, constrained, multi-objective, etc). Numerous efforts have been employed to improve the working efficiency, scalability, speed, robustness and accuracy of DE. Unlike traditional evolutionary programming (EP) and evolutionary strategies (ES), DE is based on the population members generated in the current generation with respect to randomly different members of the search space. Here, no probability based distribution (Gaussian distribution in case of EP and ES, Cauchy distribution in fast EPs) is required to generate new offspring. Numerous recent modifications have been added to DE as self-adaptive DE (SaDE)^24^, adaptive differential evolution with optional external archive (JADE)^25^, success-history based adaptive DE (SHADE)^26^, SHADE with population size reduction hybrid with semi-parameter adaptation of CMA-ES (LSHADE-SPACMA)^27^, hybrid ES-DE^28^ and others.

In this paper, a hypothesis of using a relatively new concept of iterative division to improve the exploration (*expl*) and exploitation (*expt*) operation and overcome the local optima stagnation is used^29^. Apart from this, four new modifications are added in the conventional DE to improve its overall performance. Firstly, an adaptive proportional population size reduction mechanism, inspired by GA^30^, is followed. Secondly, a reducing Weibull distributed^31^ crossover rate *CR* is introduced such that during the initial stages, the algorithm performs extensive *expl* whereas in final stages, *expt* is followed. The next modification follows the Gaussian sampling mechanism by hybridizing basic search equations to mitigate the problem of premature convergence and reinforce complementary searching capabilities^32^. Finally, instead of using a simple crossover and mutation operations, new hybridization based on grey wolf optimization (GWO)^20^ and cuckoo search (CS)^29^ are incorporated to improve the overall all performance of DE. The proposed algorithm has been named as multi-hybrid differential evolution (MHDE) algorithm. The resulting framework has been integrated with the basic DE and tested on IEEE CEC 2005^33^, CEC 2014^34^ and CEC 2017^35^ test suites, four engineering design problems and three frame design problems. The results indicate that adding additional hybridization and self-adaptivity helps in providing reliable results.

The rest of the research article is given as, “Frame design problems” section provide details about the basics of frame optimization problems. “The proposed algorithm” section describes the proposed approach, its requirement and implementation. In “Numerical examples” section, numerical results on CEC 2005 test suite, CEC 2014 and CEC 2017 benchmark problems are presented whereas in “Real-world applications I: engineering design problems” section, four engineering design problems including pressure vessel design, rolling element bearing design, tension/compression spring design and cantilever beam design are discussed. In “Real-world applications II: frame design problems” section, design of 1-bay 8-story frame, 3-bay 15-story frame and 3-bay 24 story frames are presented. Finally, in “Conclusion” section, insightful conclusions and future recommendations are unearthed.

### Frame design problems

Frame design problem is one among the most significant structural engineering design problem and has a diversified design flexibility^36^. The generalized equation for optimal frame design is given by

1 $$\begin{aligned} Find \hspace{10pt} X= [x_1, x_2,\ldots ,X_{ng}] \end{aligned}$$

2 $$\begin{aligned} to \hspace{5pt} minimize \hspace{5pt} f(X)=g(X)\times g_{penalty}(X) \end{aligned}$$

For *W* sections, *X* is the cross-sectional areas design vector, *f*(*X*) represents merit functions, *ng* is the number of design variables; *g*(*X*) is the objective function defined as the volume or weight of the frame structure; $g_{penalty}(X)$ is defined as a penalty function and is a result of constraint violations on structural response.

The frame structure weight in the form of a function *g*(*X*) is given by

3 $$\begin{aligned} g(X)=\sum _{i=1}^{nm} \gamma _i. X_i. L_i \end{aligned}$$

where *mn* is the total members making up the frame; $L_i$ is the length of the $i_{th}$ member within the frame; and $\gamma _i$ density of the material in the $i_{th}$ member.

The penalty function, $g_{penalty}(X)$ is given by^28^:

4 $$\begin{aligned} g_{penalty}(X)=(1+ \epsilon _1. v)^{\epsilon _2}, \hspace{10pt} v=\sum _{i=1}^n max [0, o_i] \end{aligned}$$

where n is the number of constraints of the design problem, $\epsilon _1$ and $\epsilon _2$ are constants based on *expl* and *expt*, and $o_i$ is the displacement or stress constraint. If $o_i$ has a positive value, the corresponding value is added to the constraint functions. These constraints consist of

Element stresses

5 $$\begin{aligned} o_i^{\sigma }=1-\Big |\frac{\sigma _i}{\sigma _i^a}\Big |\le 0, \hspace{10pt} i=1,2,\ldots ,nm \end{aligned}$$

Maximum latent displacement

6 $$\begin{aligned} v^{\Delta }=R-\frac{\Delta T}{H} \le 0 \end{aligned}$$

Inter-story displacements

7 $$\begin{aligned} v_j^d=R_I-\frac{d_j}{h_j}\le 0, \hspace{10pt} j=1,2,\ldots ,ns \end{aligned}$$

where $\sigma _i$ and $\sigma _i^a$ is the stress and allowable stress in the *i*th member respectively; $\Delta T$ is the maximum latent displacement; *ns* is the total number of stories; *R* and $d_j$ is the maximum drift index and inter-story drift respectively; *H* and $h_j$ is the height of the frame structure and story height of *j*th floor; $R_I$ represents the inter-story drift index allowed by AISC 2001^28^ and is set to 1/300. The constraints as per LRFD interactions formulas o AISC 2001 are given by

8 $$\begin{aligned} o_i^I= {\left\{ \begin{array}{ll} 1-\frac{P_u}{2\phi _c P_n}-\left( \frac{M_{ux}}{\phi _bM_{nx}+\frac{M_{uy}}{\phi _bM_{ny}}}\right) \le 0; \hspace{1pt} For \frac{P_u}{\phi _cP_n} < 0.2 \\ 1-\frac{P_u}{\phi _c P_n}-\frac{8}{9}\left( \frac{M_{ux}}{\phi _bM_{nx}+\frac{M_{uy}}{\phi _bM_{ny}}}\right) \le 0; \hspace{1pt} For \frac{P_u}{\phi _cP_n} \ge 0.2 \end{array}\right. } \end{aligned}$$

where $P_u$ and $P_n$ is the required and nominal tension or compression axial strength respectively, $\phi _c =0.9$ and $\phi _c=0.85$ are the resistance factor for tension and compression respectively, $\phi _b=0.90$ is the flexural resistance reduction factor; $M_{ux}$ and $M_{uy}$, $M_{nx}$ and $M_{ny}$ are the flexural’s required strength and flexural nominal strengths respectively in the *x* end *y* direction. For a two-dimensional structure, the value of $M_{ny}=0$.

In order to find the Euler and compression stresses, the effective length factor *K* is required. For bracing and beam members, $K=1$ and for the column members, it is calculated by using SAP2000. For a generalized case, the approximate effective length within $-1.0\%$ and $+2.0\%$ accuracy are based on Dumonteil^37^ and are given by

9 $$\begin{aligned} K= {\left\{ \begin{array}{ll} \sqrt{\frac{1.6G_AG_B+4(G_A+G_B)+7.5}{G_A+G_B+7.5}}; \hspace{5pt} For \hspace{2pt} unbraced \hspace{2pt} members \\ \frac{3G_AG_B+1.4(G_A+G_B)+0.64}{3G_AG_B+2(G_A+G_B)+1.28}; \hspace{10pt} For \hspace{2pt} braced \hspace{2pt} members \end{array}\right. } \end{aligned}$$

where $G_A$ and $G_B$ at the two end joints *A* and *B* of the column section is the stiffness ratio of two columns and girders respectively.

## The proposed algorithm

It is already known that even though a lot of new DE variants have been proposed, but it still suffers from various problems including poor *expl*, unbalanced *expl* versus *expt* operation and premature convergence^23^. Therefore, it becomes necessary to adopt changes, hybridize and add prospective modifications in the basic algorithm to overcome its inherent drawbacks and limitations. In the present work, the structure of DE is changed, and new adaptations are added in the crossover and mutation operation of the algorithm. Here GWO based equations^20^ are added in the crossover phase to improve the *expl* operation whereas mutation operation is enhanced by using CS based^29^ hybridization to enhance the *expt* operation. Apart from these modifications, the concept of iterative division is added so that considerable *expl* and exhaustive *expt* is performed towards the start whereas substantial *expt* and in-depth *expl* is performed with in certain sections towards the end^29^. The proposed MHDE is presented in the following steps:

### Initialization

The first and the foremost step, like any other algorithm, in the MHDE algorithm is the initialization phase. Here new solutions are selected randomly within the search space. The general equation is thus given by

10 $$\begin{aligned} x_{i,j} = n_{min,j} + U(0,1) \times (n_{max,j}-n_{min,j}) \end{aligned}$$

where $n_{min,j}$ and $n_{max,j}$ are the lower bounds and upper bounds and $x_{i,j}$ is *i*th solution of a *j* dimensional problem (*D*), and *U*(0, 1) is a uniform random number distributed over [0, 1].

#### Mutation operation

At each generation, DE employs crossover operation, which is controlled by a scaling factor. The target solution is achieved by different mutation strategies. The most popular strategies are given by^23^

11 $$\begin{aligned} o_i^t= x_{i}^t+ F.(x_{i}^t-x_{j}^t); \hspace{3pt} ``DE/rand/1" \end{aligned}$$

12 $$\begin{aligned} o_i^t= x_{best}^t+ F.(x_{i}^t-x_{j}^t); \hspace{3pt} ``DE/best/1" \end{aligned}$$

where $x_i^t$ and $x_j^t$ are the random solutions corresponding to the *i*th and *j*th member with *D* dimension, $o_i^t$ is the velocity corresponding to the target solution, *F* is the scaling factor, $x_{best}$ is the best solution and *t* is the current iteration. Here the equation derived from DE/rand/1 is more of an exploratory nature with increased diversity among the search agents whereas DE/best/1 has intensification properties, which promotes exploitative search around the best solution. In the proposed MHDE, both these equations are used in an adaptive manner and is explained as

For the first half of the iterations, DE/rand/1 equation is used along with GWO based equations to perform the global search operation. The scaling factor uses Lévy distribution, as discussed in subsequent subsections. Here, GWO based equations are used to perform *expt* and *expl*. This is possible because of the presence of better *expl* capabilities of GWO^20^. The new solutions generated using modified equations is thus given by

13 $$\begin{aligned} x_1=x_i-W_1\left( H_1.o_i^t-x_{i}^t\right) \end{aligned}$$

14 $$\begin{aligned} x_2=x_i-W_2\left( H_2.o_i^t-x_{i}^t\right) \end{aligned}$$

15 $$\begin{aligned} x_3=x_i-W_3\left( H_3.o_i^t-x_{i}^t\right) \end{aligned}$$

16 $$\begin{aligned} o_i^t=\frac{x_1+x_2+x_3}{3} \end{aligned}$$

where $W_1, W_2, W_3$ and $H_1,H_2,H_3$ are generated randomly from $W=2a.e_1-a$ and $H=2.e_2$, *a* is a linearly decreasing random number $\in [0,2]$ whereas $e_1$ and $e_2$ lies between [0, 1]. The whole search process consists of DE/rand/1 equation and the new GWO inspired equations, which helps the algorithm in enhancing its *expl* properties.

For the other iterative half, the DE/best/1 equation along with Gaussian random sampling is used^32^. The general equation for this phase is the same as DE/best/1 equation with an additional advantage of the Gaussian mutation to deal with the local best solution. Here, *m* new solutions are spawned and compared in accordance with $x_{best}$. If the new solution *m* is better than $x_{best}$, $x_{best}$ is replaced by the new solution. Also, it is only followed if the local best solution is not improving in a single iteration. For this strategy, the general equation is given by:

17 $$\begin{aligned} mutx_{t,d}=x_d\times (1-G(0,1)) \end{aligned}$$

where *G*(0, 1) is a random number. Apart from this modification, the whole search operation is the same as the DE/best/1 equation. The main goal is to search for potential global best solution without getting trapped in local optimal solution. The search process is followed for consecutive iterations and over the course of time, the final best solution is updated. Thus, overall helps in reinforcing complementary searching capabilities^32^ to prevent the algorithm from local optima stagnation.

#### Crossover operation

Crossover (can be arithmetic, exponential or binomial) is the next step of DE and is meant for creating the final offspring vector $x_i^t$. Here, the most commonly used binomial crossover operation is used. In this kind of crossover, each component of $x_i^t$ either comes from the mutated vector $o_i^t$ or $x_i^t$ itself, and is given as

18 $$\begin{aligned} x_i^t= {\left\{ \begin{array}{ll} o_i^t, \hspace{10pt} if (rand_k[0,1]\le CR), \hspace{3pt} j=1,2,\ldots ,n \\ x_i^t, \hspace{10pt} otherwise \end{array}\right. } \end{aligned}$$

where $rand_k[0,1] \in [0,1]$ and is continuously changed with respect to the *j*th part of the *i*th member of the population, *CR* is crossover rate and helps in controlling the extent of $o_i^t$ and $X_i^t$. This parameter is really important and helps in balancing *expl* as well as *expt* operation. In the proposed MHDE algorithm, modification has been added in the solution $x_i^t$ (used in Eq. 18). Here the solution $x_i^t$ is not the previous solution but is based on the local search equation of the CS algorithm and the general equation is given by

19 $$\begin{aligned} x_{i}^{t}=x_i^t+\epsilon \otimes \left( x_{e_1}^t-x_{e_2}^t\right) \end{aligned}$$

Here all the notations are the same as discussed in the mutation operation, apart from $\epsilon$ which is a uniformly distributed random number generated using an adaptive strategy (discussed in subsequent subsections) and lies in the range of [0, 1]. The main aim is to equally balance the local and the global search without losing the diversity among the search agents. Here mutation is performed using Eq. (19), which helps the algorithm in providing extensive search capabilities and instead of using a previous solution, a new generalized solution is used for maintaining diversity among the search agent (intensive *expt* operation). The next step is selection operation.

#### Selection operation

For any minimization process, the fitness $f(x_i^t)$ for the $x_i^t$ solution is given by Eq. (20)

20 $$\begin{aligned} x_{new}^{t+1}= {\left\{ \begin{array}{ll} x_{new} \hspace{20pt}if f(x_{new})<f(x_i^t)\\ x_i^t \hspace{30pt} otherwise \end{array}\right. } \end{aligned}$$

Here a generalized Roulette wheel selection mechanism is followed to find the final best solution. The next section deals with the various parameters of the proposed algorithm.

#### Parametric adaptation

In DE, a balanced *expl* and *expt* operation is achieved by optimizing *F* and *CR*. One among the earliest studies were conducted by^38^ where efficient values were $0<\textit{CR}<0.2$ and $0.4<\textit{F}<0.95$, while in^39^, values of $0.1<\textit{CR}<1.0$ and $0.15<\textit{F}<0.5$ were used and in^24^ self-adaptive *F* and *CR* provided better results. Overall, *CR* and $F \in [0,1]$. The parameter *F* is meant for improving the *expl* properties of DE and in present work, Lévy flights are used to imitate this operation. The Lévy flight mechanism is highly efficient and generates larger step sizes, enhancing the *expl* properties. The step size based on Lévy flights is generated as

21 $$\begin{aligned} \ F(\lambda )\sim \frac{\lambda \Gamma (\lambda )\sin (\pi \lambda /2)}{\pi }\frac{1}{s^{1+\lambda }} \hspace{2pt}(s\gg s_0\gg 0) \end{aligned}$$

where $\hspace{5pt} s=\frac{U}{|V|^1/\lambda }\hspace{2pt} U\sim N(0,\sigma ^2), \hspace{10pt}V\sim N(0,1)$ and $\sigma ^2=\bigg \{\frac{\Gamma (1+\lambda )}{\lambda \Gamma [(1+\lambda )/2]}. \frac{\sin (\pi \lambda /2)}{2^{(\lambda -1)/2}}$. Here $\lambda =1.5$ and $\Gamma$ is the gamma function. The parameter N has mean 0 and variance $\sigma ^2$ and is taken from a Gaussian distribution.

The second parameter *CR* is mainly meant for drifting the algorithm from *expl* to *expt*. Here based on *CR*, new solutions are kept if it is improved over subsequent iterations and if there is no improvement in the new generation solutions, the solutions are inspired by CS based hybridization. Though, work has been done for improving *CR* but it has been found that adding adaptive properties can provide more reliable results^24^. These conclusions pave the way for the requirement of a new distribution, which can help MHDE in a gradual transition from *expl* to *expt* without losing the global solution. Here, Weibull distribution has been used to overcome this drawback^31^. The probability distribution function is given by

22 $$\begin{aligned} CR(t)=\frac{\beta }{\eta }\Big (\frac{t-\gamma }{\eta }\Big )^{\beta -1} \hspace{5pt} e^{-(\frac{t-\gamma }{\eta })^\beta } \end{aligned}$$

where $f(t)\ge 0$, $\beta >0$, $t\ge 0\hspace{5pt} or \hspace{5pt}\gamma$, $\eta >0$, $-\infty <\gamma >\infty$. It has three main parameters namely shape ($\beta$), scale ($\eta$) and location ($\gamma$) parameter. In most of the cases, $\gamma =0$; $\beta$ helps in switching between different distributions including L-shaped distribution for $\beta \le 1$, normal distribution for $\beta =3.602$, bell-shaped for $\beta >1$ and others. For the present case, the two parameter Weibull distribution is used with $\eta$ equals to the maximum iterations and $\beta =2$. The values of Weibull distribution are taken from the literature^31^.

In the mutation phase, there is a new $\epsilon$ parameter, inspired from CS, and is meant for improving the local search capabilities of an algorithm. The parameter is adapted in accordance with the scaling factor, as in case of^35^. The general equation is given by

23 $$\begin{aligned} \varepsilon _i^{t+1}=\frac{1}{2}\times \Big (sin(2\pi \times freq \times t + \pi )\times \frac{t_{max-t}}{t_{max}} +1\Big );\hspace{5pt}if\; e_1 >0.5 \end{aligned}$$

24 $$\begin{aligned} \varepsilon _i^{t+1}=\frac{1}{2}\times \Big (sin(2\pi \times freq \times t)\times \frac{t_{max-t}}{t_{max}} +1\Big );\hspace{5pt} if\; e_1< 0.5 \end{aligned}$$

Here *freq*, is a fixed function, *t* and $t_{max}$ is the current and the maximum iterations. This parameter has been used only during the mutation operation and is intended for exploiting. Thus, three parameters are there to improve overall stability of the proposed MHDE algorithm.

#### Population adaptation

Population-based algorithms require an initial set of random solutions to start their search operation. Population decides three things, total spawned solutions, maximum function evaluations and complexity of an algorithm. A static population keeps the total function evaluations constant, whereas an adaptive decreasing population can reduce them significantly. The concept of adaptive population was formulated in^40^ and was extended to GA^30^. In^40^ with an increasing solution fitness, the population was decreased whereas for decreasing solution fitness, the population was increased. The major drawback of this formulation was the formation of new clones of existing solutions, paving the way for reduced performance. In^30^, an opposite adaptation was followed by reducing the population if the best fitness in increasing. For a multimodal problem, the algorithm should be able to optimize large landscapes. Initially, if there is a large population size, the fitness will be very high. The algorithm will explore the search space and, over the course of iterations, starts moving toward some random direction. Here because of the higher fitness, chances are there that new solutions are in the same direction and hence population size can be reduced^30^. This new population helps to find potential solutions without losing the final best solution. Also, with increase in the iterations, the variation in solution quality is marginal and hence using a smaller population provide many reliable results. This is because, each member in a small population has a higher probability of becoming the local best and eventually the global best solution. The mathematical equation deduced by^30^ is given as

25 $$\begin{aligned} N_{t+1}= {\left\{ \begin{array}{ll} (1-\Delta f_t^{best})N_t, \hspace{10pt} if \; \Delta f_t^{best}\le \Delta f_{max}^{best}\\ (1-\Delta f_{max}^{best})N_t, \hspace{10pt} if \; \Delta f_t^{best}> \Delta f_{max}^{best}\\ {min}_{N}, \hspace{10pt} if \; N_{t+1} <{min}_{N} \end{array}\right. } \end{aligned}$$

Here *N* is the population for the $t_{th}$ generation, $\Delta f_t^{best}$ is given by $\Big (\frac{f_{t-1}^{best}-f_{t-2}^{best}}{|f_{t-2}^{best}|}\Big )$ is change in the best fitness, $\Delta f_{max}^{best}$ is the threshold value. It should be noted that a minimum fitness must be defined so that all the negative effects of minimal population are minimized.

## Numerical examples

The proposed MHDE algorithm is analysed for numerical benchmark datasets and compared with respect to other recent hybrid algorithms. Two benchmark sets have been used, namely classical benchmark problems from CE C2005 test suite^33^ and CEC2014 test suite^25^. For comparison on CEC 2005, the major algorithms used are JADE^25^, Evolution strategy based on covariance adaptation (CMA-ES)^21^, SaDE^41^, a sine cosine crow search algorithm (SCCSA)^42^, extended GWO (GWO-E)^20^, fractional-order calculus-based FPA (FA-FPO)^43^, SHADE^26^ and LSHADE-SPACMA^21^. On the other hand for CEC 2014 benchmark problems, blended biogeography-based optimization (B-BBO)^44^, laplacian BBO (LX-BBO)^44^, random walk GWO (RW-GWO)^44^, population-based incremental learning (PBIL)^45^, improved symbiotic organisms search (ISOS)^46^, variable neighbourhood BA (VNBA)^45^, chaotic cuckoo search (CCS)^45^, and improved elephant herding optimization (IMEHO)^45^ are used.

**Table 1:** Parameter settings of different algorithms.

Algorithm	Parameters
CCS^45^	$(p_a)$ = 0.25
JADE^25^	$F=0.5$; $CR=0.9$; $1/c=[5,20]$; $p=[0.05,0.20]$
SaDE^25^	*F*, *CR* = self adaptive
ISOS^46^	$q \in [1,100]\%$, $r \in [0,1]$
PBIL^45^	(*LR*) = 0.1; $(p_m)$ = 0.02
VNBA^45^	(*A*) = 0.5; (*r*) = 0.5
SCCSA^42^	$e_1, e_2, e_3 = [0,1]$
GWO-E^20^	$\mathbf {\alpha }$ = Linearly decreasing from 2 to 0
FA-FPO^43^	$\alpha = [0.1,1]$, $S=adaptive$
B-BBO^44^	$H=1$; $I=1$
FO-FPA^43^	$\alpha = [0.1,1]$; $S=adaptive$; $r=2$ or 4 or 8
IMEHO^45^	(*w*)= Linearly decreasing [0.9,0.2]; $\alpha$ $\epsilon$ [0,1]; $(p_c)$ = 0.05
SHADE^21^	$P_{best}=0.1$, $ARC\; rate$ = 2
LX-BBO^44^	$H=1$; $I=1$
CMA-ES^21^	$n=\mu =10$; $\lambda =40$
RW-GWO^44^	$\mathbf {\alpha }$ = Linearly decreased from 2 to 0
LSHADE-SPACMA^21^	*c*=0.8, $P_{best}=0.11$, *ARC* *rate* = 1.4, *FCP* = 0.5
Proposed MHDE	All parameters (*F*, *CR* and $\epsilon$) are adaptive

For all test categories, the parametric details for all the algorithms under comparison is given in Table 1. Apart from the basic parameters, a $population size = 50$, $D = 30$ and total of 51 runs is used for evaluation. For CEC2005, the total function evaluations are taken as 15, 000 whereas for CEC2014, the maximum function evaluations are set^34^ as $10^4$ $\times$ *D*. The results for both the test cases are evaluated as mean error and standard deviation (std)^34^. It must be noted that the bold values in all the tables signifies the best algorithm corresponding to that particular problem.

For statistical testing, two statistical tests, namely Friedman rank (f-rank) and Wilcoxon’s rank-sum tests^47^ are used. The results are presented as ranks found by p-values at $5\%$ level of significance. For every test function, the statistical results are presented as *win*(*w*)/*loss*(*l*)/*tie*(*t*). Here *win*(*w*) is the situation where the test algorithm is better than MHDE algorithm, the *loss*(*l*) scenario on the other hand, is the situation where the algorithm is worse than MHDE algorithm and “-” sign means *tie*(*t*) and it denotes that both the algorithms under consideration are either statistically similar or irrelevant in accordance to each other^47^. Apart from that, the f-rank is calculated for every function and an average of all the ranks is presented. In the next subsections, analysis on CEC 2005 benchmark problems is presented.

### Classical benchmarks

A comparison of MHDE is performed with respect to the well-known variants of DE including JADE, SaDE, SHADE and LSHADE-SPACMA as well as some recently introduced algorithms including GWO-E, SCCSA, FA-FPO and CMA-ES as given by Table 2. Here $G_1-G_7$ are unimodal functions (for testing *expt* capabilities), $G_8-G_{12}$ are multi-modal functions (for a balanced *expl* and *expt* operation), and $G_{13}-G_{15}$ are fixed dimension (convergence analysis), testing the effectiveness and consistency of the MHDE algorithm for finding the optimal solution. These test functions are defined in^48^ and are not explicitly discussed in the present paper.

**Table 2:** Simulation results for CEC 2005 benchmarks.

Function		JADE	SaDE	GWO-E	SCCSA	FA-FPO	CMA-ES	SHADE	LSHADE-	MHDE
		^25^	^25^	^20^	^42^	^43^	^21^	^21^	SPACMA^21^	
*G*1	Mean	1.80E−60	4.50E−20	3.92E−67	9.22E−69	1.51E−184	1.42E−18	1.42E−09	2.23E−01	2.93E−254
	Std	8.40E−60	6.90E−20	1.11E−66	3.81E−68	0.00E+00	3.13E−18	3.09E−09	1.48E−01	0.00E+00
	f-rank	5	6	4	3	2	7	8	9	1
*G*2	Mean	1.80E−25	1.90E−14	4.31E−36	8.25E−41	**5.04E−93**	2.98E−07	8.70E−03	2.11E+01	2.78E−18
	Std	8..8E−25	1.05E−14	6.57E−36	4.19E−40	**3.47E−93**	1.78E+00	2.13E−02	9.57E+00	4.76E−18
	f-rank	4	6	3	2	1	7	8	9	5
*G*3	Mean	5.70E−61	9.00E−37	3.75E−37	4.31E−13	1.23E−183	1.59E−05	1.54E+01	8.87E+01	**1.07E−255**
	Std	2.70E−60	5.43E−36	1.36E−36	2.83E−30	0.00E+00	2.21E−05	9.94E+00	4.72E+01	**0.00E+00**
	f-rank	3	5	4	6	2	7	8	9	1
*G*4	Mean	8.20E−24	7.40E−11	2.39E−25	2.15E−17	9.97E−93	2.01E−06	9.79E−01	2.11E+00	**2.07E−147**
	Std	4.00E−23	1.82E−10	6.80E−25	1.06E−16	7.31E−93	1.25E−06	7.99E−01	4.92E−01	**6.93E−147**
	f-rank	4	6	3	5	2	7	8	9	1
*G*5	Mean	8.00E−02	2.10E+01	2.65E+01	5.90E+00	2.89E+01	3.67E+01	2.44E+01	2.88E+01	**0.00E+00**
	Std	5.60E−01	7.80E+00	5.19E−01	9.13E−01	1.72E−02	3.34E+01	1.12E+01	8.24E−01	**0.00E+00**
	f-rank	2	4	6	3	8	9	5	7	1
*G*6	Mean	2.90E+00	9.30E+02	2.65E+01	4.14E−08	5.88E+00	6.83E−19	5.31E−10	2.48E−01	**1.20E−21**
	Std	1.20E+00	1.80E+02	5.19E−01	5.22E−08	5.86E−01	6.71E−19	6.35E−10	1.13E−01	**2.68E−21**
	f-rank	6	9	8	4	7	2	3	5	1
*G*7	Mean	6.40E−04	4.80E−03	9.90E−04	1.33E−03	1.13E−04	2.75E−02	2.35E−02	4.70E−03	**5.54E−06**
	Std	2.50E−04	1.20E−03	8.37E−04	1.72E−03	8.94E−04	7.90E−03	8.80E−03	1.90E−03	**4.47E−06**
	f-rank	3	7	4	5	2	9	8	6	1
*G*8	Mean	1.00E−04	1.20E−03	**0.00E+00**	5.46E+00	**0.00E+00**	2.53E+01	8.53E+00	6.75E+01	**0.00E+00**
	Std	6.00E−05	6.50E−04	**0.00E+00**	5.62E+00	**0.00E+00**	8.55E+00	2.19E+00	1.00E+01	**0.00E+00**
	f-rank	2	3	1	4	1	6	5	7	1
*G*9	Mean	8.20E−10	2.70E−03	5.58E−15	**8.88E−16**	**8.88E−16**	1.55E+01	3.95E−01	3.93E−02	**8.88E−16**
	Std	6.90E−10	5.10E−04	1.67E−15	9.36E−32	**0.00E+00**	7.92E+00	5.86E−01	1.51E−02	**0.00E+00**
	f-rank	3	4	2	1	1	7	6	5	1
*G*10	Mean	9.90E−08	7.80E−04	**0.00E+00**	3.33E−02	**0.00E+00**	5.76E−15	4.80E−03	8.94E−01	**0.00E+00**
	Std	6.00E−07	1.20E−03	**0.00E+00**	4.56E−02	**0.00E+00**	6.18E−15	7.70E−03	1.07E−01	**0.00E+00**
	f-rank	3	4	1	6	1	2	5	7	1
*G*11	Mean	4.60E−17	1.90E−05	1.98E−02	1.34E−02	8.32E−01	2.87E−16	3.46E−02	8.18E−04	**2.74E−18**
	Std	1.90E−16	9.20E−06	1.01E−02	1.60E−02	1.78E−01	5.64E−16	8.75E−02	1.00E−03	**4.76E−18**
	f-rank	2	4	7	6	9	3	8	5	1
*G*12	Mean	2.00E−16	6.10E−05	2.50E−01	2.01E−02	2.94E+00	3.66E−04	7.32E−04	1.02E−02	**1.35E−20**
	Std	6.50E−16	2.00E−05	1.63E−01	7.23E−02	1.59E−01	2.00E−03	2.80E−03	1.03E−02	**2.51E−20**
	f-rank	2	3	8	7	9	4	5	6	1
*G*13	Mean	3.00E+00	3.00E+00	3.00E+00	3.00E+00	3.00E+00	8.40E+00	3.00E+00	3.00E+00	3.00E+00
	Std	1.10E−15	3.00E−15	4.96E−04	8.93E−05	3.13E−09	2.05E+01	1.87E−15	1.25E−15	**4.57E−16**
	f-rank	2	5	8	7	6	9	4	3	1
*G*14	Mean	−3.86E+00	−3.86E+00	−3.86E+00	−3.86E+00	−3.01E−01	−3.86E+00	−3.86E+00	−3.86E+00	−1.89E+00
	Std	**0.00E+00**	3.10E−15	4.16E−06	9.29E−06	2.25E−16	2.70E−15	2.69E−15	2.70E−15	1.56E−15
	f-rank	1	5	6	7	2	4	3	4	8
*G*15	Mean	−3.31E+00	−3.31E+00	−3.26E+00	−3.26E+00	−3.29E+00	−3.29E+00	−3.27E+00	−3.28E+00	−1.16E+00
	Std	3.60E−02	2.80E−02	7.50E−02	**6.00E−02**	1.97E−02	5.35E−02	5.99E−02	5.70E−02	6.28E−17
	f-rank	7	6	5	1	1	4	2	3	8
w/l/t		3/12/0	2/13/0	3/11/1	3/11/1	3/10/2	2/13/0	2/13/0	2/13/0	
Overall f-rank		56	75	70	67	64	67	86	94	33
Average f-rank		3.73	5	4.66	4.46	4.26	4.46	5.73	6.26	2.20
Overall f-rank		2	6	5	4	3	4	7	8	1

The results are presented as mean and std values for 30 dimension size. For $G_1$, $G_3$, $G_4$, $G_5$, $G_7$, $G_{11}$, $G_{12}$ and $G_{13}$ functions, the algorithm performs better in comparison to others. For $G_8$ and $G_{10}$ functions, GWO-E, FA-FPO and the proposed MHDE performs equivalently whereas for function $G_9$, SCCSA, FA-FPO have equivalent results with respect to MHDE. Apart from that, JADE is found to be better for $G_{14}$ and SCCSA for $G_{15}$ function. The statistical results show that MHDE converges to better solutions than JADE, SaDE, SHADE and LSHADE-SPACMA and others, which indicate that MHDE is an excellent algorithm.

Furthermore, the Friedman f-rank test and Wilcoxon rank sum tests are conducted to analyse the results of MHDE with respect to other algorithms for 51 individual trials for each function. Taking JADE versus MHDE as an example, *w*/*l*/*t* ratio and average f-rank of MHDE is better than JADE, it means that MHDE is significantly better than JADE at the 5% significance level or 95% level of confidence. Thus overall, ranking analysis results between DE variants, MHDE and other algorithms show that the proposed MHDE is significantly better.

**Table 3:** Sensitivity analysis of parametric adaptations.

Function		Adaptive *F* and *CR*	Adaptive crossover	Adaptive *N* and mutation	Adaptive *CR* and mutation	Adaptive mutation and *F* & *N*
$G_1$	Mean	577E−321	9.01E−256	1.19E−249	1.10E−257	3.04E−240
	Std	2.94E−319	0.00E+00	0.00E+00	0.00E+00	000E+00
$G_2$	Mean	4.54E−167	1.78E−128	2.57E−125	2.78E−127	2.39E−122
	Std	1.25E−165	8.90E−128	6.53E−125	1.93E−126	1.45E−121
$G_3$	Mean	4.26E−314	3.46E−255	4.16E−247	3.64E−256	1.15E−241
	Std	217E−312	0.00E+00	0.00E+00	0.00E+00	0.00E+00
$G_4$	Mean	1.61E−163	9.92E−130	2.87E−125	4.61E−128	766E−122
	Std	0.00E+00	2.89E−129	1.78E−124	2.27E−128	4.00E−121
$G_5$	Mean	0.00E+00	0.00E+00	0.00E+00	0.00E+00	0.00E+00
	Std	000E+00	0.00E+00	0.00E+00	0.00E+00	0.00E+00
$G_6$	Mean	9.06E−12	4.46E−20	9.31E−12	4.93E−20	1.53E−11
	Std	1.42E−11	9.86E−20	1.62E−11	1.11E−19	4.38E−11
$G_7$	Mean	1.35E−05	1.76E−05	1.73E−05	1.48E−05	1.68E−05
	Std	1.38E−05	1.42E−05	1.48E−05	9.37E−06	1.58E−05
$G_8$	Mean	0.00E+00	0.00E+00	0.00E+00	0.00E+00	0.00E+00
	Std	0.00E+00	0.00E+00	000E+00	0.00E+00	0.00E+00
$F_{9}$	Mean	8.88E−16	8.88E−16	8.88E−16	8.88E−16	8.88E−16
	Std	8.81E−16	8.88E−16	888E−16	8.88E−16	8.88E−16
$G_{10}$	Mean	0.00E+00	0.00E+00	000E+00	0.00E+00	0.00E+00
	Std	0.00E+00	0.00E+00	0.00E+00	0.00E+00	0.00E+00
$G_{11}$	Mean	1.00E−09	7.41E−17	1.11E−09	9.17E−17	6.98E−10
	Std	1.70E−09	1.73E−16	2.27E−09	2.10E−16	1.14E−09
$G_{12}$	Mean	4.08E−10	2.92E−19	1.57E−10	4.44E−19	6.04E−10
	Std	9.92E−10	4.22E−19	3.30E−10	1.12E−18	1.60E−09

Sensitivity analysis is done to check how the newly introduced parameters affect the performance and efficiency of MHDE. In Table 3, five different adjustments are made in the proposed modifications, and two statistical indicators (mean and std) are used to describe it. The same set of parameters and function evaluations are used as used for CEC 2005 benchmark testing. The improved crossover operation helps in performance enhancement for unimodal functions and hence leading to better *expt* properties. Addition of adaptive *F* helps in improving the global search capabilities and hence provides better *expl*. Adding adaptivity in *CR* and mutation operation helps in enhancing the accuracy for multi-modal functions, whereas adaptive population size *N* helps in reducing the function evaluations. Furthermore, the results of MHDE at different parameters are all better than JADE, SaDE and other hybrid versions of DE. To sum up, the performance of MHDE is robust and excellent. To further validate the superiority of MHDE with respect to some recently introduced algorithms, CEC 2014 benchmark test suite is used and has been explained in details in the next subsection.

### CEC 2014 benchmarks

For CEC 2014 benchmarks, proposed MHDE algorithm and eight recently introduced hybrid algorithms have been selected for comparison. All of these algorithms are enhanced versions of new population-based algorithms and are B-BBO^44^, LX-BBO^44^, RW-GWO^44^, PBIL^45^, ISOS^46^, VNBA^45^, CCS^45^, and IMEHO^45^. The mean error and std values of all of these variants on 30 dimension problems are listed in Table 4.

**Table 4:** Statistical results for CEC 2014 benchmark functions.

Functions	Error	LX-BBO	B-BBO	RW-GWO	ISOS	PBIL	VNBA	IMEHO	CCS	MHDE
		^49^	^49^	^44^	^46^	^45^	^50^	^45^	^51^	
$G_1$	Mean	1.01E+07	6.50E+06	8.02E+06	9.82E+05	3.42E+08	2.43E+08	2.37E+06	1.46E+08	**7.06E+05**
	Std	1.01E+07	1.30E+06	3.31E+06	7.05E+05	1.09E+08	5.93E+07	4.32E+06	3.27E+07	**2.73E+05**
	f-rank	6	4	5	2	9	8	3	7	1
$G_2$	Mean	5.34E+04	2.35E+04	2.23E+05	**5.27E+00**	4.08E+10	1.92E+10	5.49E+03	2.60E+09	1.00E+10
	Std	2.14E+04	9.99E+03	5.51E+05	**1.72E+01**	3.39E+09	4.23E+09	4.87E+03	5.22E+08	0.00E+00
	f-rank	4	3	5	1	9	8	2	6	7
$G_3$	Mean	1.63E+04	6.03E+03	3.16E+02	4.79E+02	9.19E+04	2.93E+04	1.41E+02	2.70E+03	**2.65E−01**
	Std	1.70E+04	3.15E+03	4.34E+02	6.24E+02	1.75E+04	1.39E+04	1.58E+02	7.74E+04	**2.10E−01**
	f-rank	7	6	3	4	9	8	2	5	1
$G_4$	Mean	9.99E+01	1.02E+02	3.41E+01	5.98E+01	3.43E+03	1.60E+03	1.24E+02	3.22E+02	**2.87E+01**
	Std	2.84E+01	3.13E+01	1.80E+01	3.57E+01	7.56E+02	3.63E+02	4.77E+01	4.09E+01	3.34E+01
	f-rank	4	5	2	3	9	8	6	7	1
$G_5$	Mean	**3.06E+00**	3.74E+00	2.05E+01	2.03E+01	2.10E+01	2.10E+01	2.10E+01	2.10E+01	2.04E+01
	Std	7.86E−01	4.91E−01	7.46E−02	6.67E−02	5.56E−02	5.43E−02	5.99E−02	8.81E−02	7.11E−02
	f-rank	1	2	5	3	9	8	7	6	4
$G_6$	Mean	1.70E+01	1.99E+01	9.84E+00	1.05E+01	3.80E+01	3.30E+01	1.20E+01	2.50E+01	**8.66E+00**
	Std	3.12E+00	2.70E+00	3.49E+00	2.39E+00	1.16E+00	2.58E+00	2.72E+00	2.00E+00	1.64E+00
	f-rank	5	6	2	3	9	8	4	7	1
$G_7$	Mean	1.75E−01	7.81E−02	2.53E−01	1.56E−02	3.40E+02	1.11E+02	**0.00E+00**	2.30E+01	9.31E−04
	Std	8.56E−02	4.44E−02	1.43E−01	1.83E−02	2.74E+01	1.81E+01	1.19E−01	3.52E+00	3.29E−04
	f-rank	5	4	6	3	9	8	1	7	2
$G_8$	Mean	5.53E+01	**4.71E−01**	4.38E+01	1.47E+01	3.00E+02	1.74E+02	3.30E+01	2.90E+02	5.73E+00
	Std	3.78E+02	6.79E−01	8.48E+00	3.34E+00	1.03E+01	1.61E+01	9.19E+00	2.23E+01	2.90E+00
	f-rank	6	1	5	3	9	7	4	8	2
$G_9$	Mean	7.66E+01	9.11E+01	**6.33E+01**	2.56E+02	3.70E+02	2.50E+02	3.20E+01	2.90E+02	1.44E+02
	Std	1.61E+01	1.54E+01	1.30E+01	1.34E+01	1.69E+01	2.03E+01	1.15E+01	2.38E+01	1.23E+01
	f-rank	3	4	1	7	9	6	2	8	5
$G_{10}$	Mean	1.25E+04	6.68E+03	9.61E+02	1.78E+03	6.26E+03	3.50E+03	2.26E+03	8.55E+03	**1.82E+01**
	Std	1.16E+02	4.58E+02	2.72E+02	4.09E+01	3.05E+02	3.47E+02	5.72E+02	4.91E+02	2.47E+01
	f-rank	9	7	2	3	6	5	4	8	1
$G_{11}$	Mean	1.23E+04	6.71E+03	2.68E+03	**1.48E+03**	7.10E+03	6.80E+03	2.86E+03	8.83E+03	1.97E+03
	Std	3.41E+02	5.17E+02	3.68E+02	4.54E+02	2.97E+02	3.79E+02	5.38E+02	5.50E+02	3.40E+02
	f-rank	9	5	3	1	7	6	4	8	2
$G_{12}$	Mean	**1.11E−02**	1.11E−02	5.45E−01	3.55E−01	1.00E+01	1.00E+01	1.00E+01	1.00E+01	2.48E−01
	Std	1.75E−18	1.75E−18	1.66E−01	5.73E−02	3.38E−01	3.51E−01	5.26E−01	1.09E+00	1.01E−01
	f-rank	1	1	4	3	7	6	8	5	2
$G_{13}$	Mean	6.55E−01	6.78E−01	2.80E−01	3.77E−01	0.00E+00	0.00E+00	0.00E+00	**0.00E+00**	2.34E−01
	Std	1.56E−01	7.98E−02	6.30E−02	7.10E−02	2.56E−01	3.64E−01	6.25E−02	1.76E−01	5.01E−02
	f-rank	8	9	6	7	2	3	4	1	5
$G_{14}$	Mean	6.20E−01	3.93E−01	4.23E−01	2.71E−01	1.00E+02	6.00E+01	**0.00E+00**	1.00E+01	1.77E−01
	Std	2.96E−01	1.55E−01	2.15E−01	5.12E−02	1.16E+01	1.22E+01	9.85E−02	1.88E+00	2.16E−02
	f-rank	6	4	5	3	9	8	1	7	2
$G_{15}$	Mean	1.55E+01	1.88E+01	8.81E+00	1.06E+01	6.84E+05	2.39E+03	**0.00E+00**	8.00E+01	6.64E+00
	Std	5.49E+00	5.64E+00	1.51E+00	3.71E+00	2.85E+05	1.22E+03	1.35E+00	3.03E+01	1.75E+00
	f-rank	5	6	3	4	9	8	1	7	2
w/l/t		**6/24/0**	**5/25/0**	**2/28/0**	**3/27/0**	**1/29/0**	**1/29/0**	**8/22/0**	**3/27/0**	
Overall f-rank		173	164	107	119	221	193	112	188	63
Average f-rank		5.76	5.46	3.56	3.96	7.36	6.43	3.73	6.26	2.10
f-rank		6	5	2	4	9	8	3	7	1

Functions	Error	LX-BBO	B-BBO	RW-GWO	ISOS	PBIL	VNBA	IMEHO	CCS	MHDE
		^49^	^49^	^44^	^46^	^45^	^50^	^45^	^51^	
$G_{16}$	Mean	1.08E+01	1.06E+01	1.03E+01	9.21E+01	2.00E+01	2.00E+01	2.00E+01	2.00E+01	**9.89E+00**
	Std	5.84E−01	6.25E−01	6.11E−01	7.31E−01	2.12E−01	3.66E−01	7.64E−01	1.75E−01	0.4827E−01
	f-rank	4	3	2	9	6	7	8	5	1
$G_{17}$	Mean	1.49E+06	1.27E+06	5.71E+05	1.75E+05	9.74E+06	2.53E+06	**7.69E+04**	1.15E+07	9.66E+04
	Std	9.34E+05	5.46E+05	4.10E+05	1.64E+05	2.79E+06	3.34E+06	8.38E+04	4.59E+06	5.16E+04
	f-rank	6	5	4	3	8	7	1	9	2
$G_{18}$	Mean	2.89E+03	8.22E+02	6.52E+03	3.89E+03	6.16E+08	1.66E+08	3.30E+03	1.10E+08	**2.15E+02**
	Std	4.27E+03	1.00E+03	4.63E+02	5.15E+03	1.68E+08	1.03E+08	3.52E+03	4.66E+07	6.38E+01
	f-rank	3	2	6	5	9	8	4	7	1
$G_{19}$	Mean	5.19E+03	7.81E+03	1.14E+01	7.79E+01	1.90E+02	1.20E+02	1.05E+01	4.00E+01	**6.01E+00**
	Std	5.67E+03	4.67E+03	2.03E+00	1.78E+00	3.42E+01	3.82E+01	1.74E+00	5.91E+00	5.40E−01
	f-rank	8	9	3	5	7	6	2	4	1
$G_{20}$	Mean	2.61E+04	1.62E+04	6.27E+02	4.98E+03	3.59E+04	1.69E+04	2.10E+02	1.03E+06	**9.12E+01**
	Std	1.57E+04	4.11E+03	1.12E+03	3.40E+03	1.77E+04	6.57E+03	8.17E+01	9.05E+05	2.83E+01
	f-rank	7	5	3	4	8	6	2	9	1
$G_{21}$	Mean	1.11E+06	1.22E+06	2.58E+05	8.90E+04	2.52E+06	2.30E+06	2.72E+04	5.66E+06	**1.75E+04**
	Std	7.95E+05	7.96E+05	1.76E+05	1.07E+05	1.19E+06	1.35E+06	1.83E+04	2.73E+06	1.06E+04
	f-rank	5	6	4	3	8	7	2	9	1
$G_{22}$	Mean	1.88E+03	1.68E+02	2.08E+02	2.75E+02	1.02E+03	8.40E+02	2.10E+02	1.34E+03	**1.26E+02**
	Std	2.03E+02	2.47E+02	2.08E+02	1.45E+02	1.88E+02	1.28E+02	1.01E+02	1.88E+02	5.60E+01
	f-rank	9	2	3	5	7	6	4	8	1
$G_{23}$	Mean	4.11E+02	3.43E+02	3.15E+02	3.15E+02	6.00E+02	3.90E+02	3.20E+02	3.50E+02	**2.00E+02**
	Std	6.43E+01	2.84E+01	2.77E−01	1.60E+01	6.70E+01	2.47E+01	4.78E−01	7.66E+00	0.00E+00
	f-rank	8	5	2	3	9	7	4	6	1
$G_{24}$	Mean	1.47E+04	3.41E+04	2.00E+02	2.00E+02	4.00E+02	2.30E+02	2.40E+02	2.20E+02	**2.00E+02**
	Std	8.37E+03	2.35E+04	3.04E−03	1.50E−03	1.42E+01	2.53E+01	6.46E+00	2.51E+00	8.21E−04
	f-rank	8	9	2	3	7	5	6	4	1
$G_{25}$	Mean	5.29E+02	6.53E+02	2.04E+02	2.00E+02	2.40E+02	2.10E+02	2.10E+02	2.20E+02	**2.00E+02**
	Std	4.36E+01	6.01E+01	1.18E+00	8.07E−01	6.00E+00	1.07E+01	2.08E+00	4.44E+00	0.00E+00
	f-rank	8	9	3	2	7	5	4	6	1
$G_{26}$	Mean	**2.12E+00**	3.64E+01	1.00E+02	1.00E+02	1.00E+02	1.00E+02	1.00E+02	1.00E+02	1.31E+02
	Std	3.46E+00	5.62E+01	7.36E−02	9.55E−02	2.09E−01	4.30E−01	6.00E−02	2.06E−01	4.66E+01
	f-rank	1	2	8	7	5	6	3	4	9
$G_{27}$	Mean	**1.95E+02**	3.04E+02	4.09E+02	5.43E+02	1.08E+03	1.29E+03	5.80E+02	5.30E+02	2.00E+02
	Std	1.04E+02	1.60E+02	6.09E+00	1.36E+02	2.80E+02	3.30E+01	1.41E+02	7.92E+01	0.00E+00
	f-rank	1	3	4	6	7	8	9	5	2
$G_{28}$	Mean	1.94E+03	2.12E+03	4.34E+02	9.68E+02	1.39E+03	1.68E+03	9.70E+02	1.44E+03	**2.00E+02**
	Std	1.04E+02	4.44E+02	8.45E+00	4.12E+01	1.33E+02	2.33E+02	2.44E+02	9.46E+02	0.00E+00
	f-rank	8	9	2	3	5	7	4	6	1
$G_{29}$	Mean	1.98E+07	3.09E+07	2.14E+02	5.70E+05	5.70E+06	7.47E+06	1.21E+03	1.20E+06	**2.00E+02**
	Std	3.95E+06	6.91E+06	2.37E+00	2.14E+06	3.33E+06	1.20E+06	2.16E+02	7.03E+05	0.00E+00
	f-rank	8	9	2	4	7	6	3	5	1
$G_{30}$	Mean	6.95E+06	1.38E+07	6.69E+02	2.38E+05	1.49E+05	1.89E+05	4.08E+03	7.67E+04	**2.00E+02**
	Std	1.03E+07	1.08E+07	2.14E+02	1.10E+03	5.55E+04	1.03E+05	1.44E+03	3.37E+04	3.44E−05
	f-rank	8	9	2	7	5	6	3	4	1
w/l/t		**6/24/0**	**5/25/0**	**2/28/0**	**3/27/0**	**1/29/0**	**1/29/0**	**8/22/0**	**3/27/0**	
Overall f-rank		173	164	107	119	221	193	112	188	63
Average f-rank		5.76	5.46	3.56	3.96	7.36	6.43	3.73	6.26	2.10
f-rank		6	5	2	4	9	8	3	7	1

Here, the results by comparing the difference between obtained solution and the desired best solution are found. If the difference becomes less than $10^{-8}$, the error is treated as zero. From Table 4, it is found that MHDE performs better than all other algorithms under consideration. Here out of three uni-modal functions ($G_1-G_3$), MHDE performs better for two among all other algorithms showing superior capability in finding global solution. This further shows that the algorithm has better *expl* properties. Among multi-modal function ($G_4-G_{10}$), MHDE performs better for three functions among all the variants and for rest of the functions it is either ranked second or third. This again shows the superior performance of MHDE for local optima avoidance. For hybrid benchmarks ($G_{11}-G_{20}$) and composite benchmarks ($G_{21}-G_{30}$), MHDE is found to be the best among all other algorithms. This further proves the capability of MHDE in balancing the *expl* and *expt* operation to achieve global best solution. Overall, MHDE is ranked first, RW-GWO is ranked second and IMEHO is ranked third among all the other algorithms under comparison. In the next section, MHDE is used for design of frame structures.

### CEC 2017 benchmarks

For a comprehensive evaluation of the proposed MHDE algorithm in comparison to MH algorithms, the SaDE^35^, SHADE^52^, JADE^35^, CV1.0^29^, $CV_{new}$^53^, MVMO^35^, and CS^17^ algorithms have been utilized with 51 run and 100 population size. In order to have a fair comparison, a maximum function evaluations as $10,000 \times D$ is used where $D = 30$ is the dimension size. The algorithms used for comparison are highly competitive and have proved their worthiness for various CEC competitions. A rank-sum test (in terms of *w*/*l*/*t*) and f-test at 0.05 level of significance^47^ is done to evaluate the performance of MHDE, along with experimental mean error and standard deviation. The mean error is evaluated by calculating the difference between the obtained values and the global optimum of that problem. From the results in Table 5, the following observations are made. For the first case of unimodal problems, $H_1$, $H_2$ and $H_3$, SHADE, JADE, SaDE, MVMO, and LSHADE give highly efficient results; $CV_{new}$, CV1.0, CS and MHDE have similar performance and SHADE performed the best for these problems. For the multimodal problems, $H_4$ to $H_{10}$, SHADE, MVMO, JADE and SaDE have similar performance and LSHADE gave the best results. For hybrid problems, $H_{11}$ to $H_{20}$, CS, $CV_{new}$, CV1.0, were better than the DE variants, and MHDE was found to be the best among others. For $H_{21}$ to $H_{30}$ composite problems, MHDE gives the best performance and is the most significant algorithm among all others under comparison. The results in the last line of Table 5, provides statistical p-values and f-rank, and it is found that with respect to MHDE, LSHADE gives better performance for 16 problems, SHADE for 14 problems, SaDE for 10 problems, JADE for 12 problems, MVMO for 15 problems, $CV_{new}$ for 6 problems.

Overall comparison shows that MHDE is better than other algorithms for most of the hybrid and composite problems and has poor performance over unimodal and multimodal problems. This further proves the significance of MHDE over statistical and experimental results, for challenging optimization problems.

**Table 5:** Statistical results for CEC 2017 benchmark problems.

Function		SaDE^35^	JADE^35^	SHADE^52^	LSHADE^54^	MVMO^35^	CV1.0^29^	$CV_{new}$^53^	CS	MHDE
$H_1$	Mean	1.212E+03	5.231E−14	**0.000E+00**	**0.000E+00**	1.331E−05	1.000E+10	1.000E+10	1.000E+10	1.000E+10
	Std	(1.972E+03)	(2.512E−14)	(0.000E+00)	(0.000E+00)	(5.602E−06)	(0.000E+00)	(0.000E+00)	(0.000E+00)	(0.000E+00)
	rank	+	+	+	+	+	=	=	=	
	f-rank	4	2	1	1	3	5	5	5	5
$H_3$	Mean	2.711E+02	1.774E+04	** 0.000E+00**	**0.000E+00**	5.303E−07	1.954E+04	8.713E+03	2.525E+05	4.093E+03
	Std	(8.282E+02)	(3.701E+04)	(0.000E+00)	(0.000E+00)	(1.094E−07)	(6.273E+03)	(4.083E+03)	(3.024E+04)	(1.504E+03)
	rank	+	−	+	+	+	−	−	−	
	f-rank	5	4	3	2	1	8	7	9	6
$H_4$	Mean	8.923E+01	4.962E+01	5.683E+01	8.184E+01	3.583E+01	1.163E+02	**2.673E+01**	1.285E+02	4.344E+01
	Std	(4.211E+01)	(4.713E+01)	(8.801E+00)	(4.083E+01))	(3.662E+01)	(6.273E+03)	(5.924E+00)	(2.444E+01)	(2.713E+01)
	rank	−	−	−	−	+	−	+	−	
	f-rank	6	5	4	7	3	8	1	9	2
$H_5$	Mean	9.232E+01	5.422E+01	3.284E+01	**1.223E+01**	8.074E+01	3.412E+02	2.394E+02	4.864E+02	2.133E+02
	Std	(1.863E+01)	(8.804E+00)	(5.033E+00)	(2.042E+00)	(1.643E+01)	(8.023E+01)	(3.803E+01)	(4.665E+01)	(2.344E+01)
	rank	+	+	+	+	+	−	−	−	
	f-rank	2	5	6	1	4	8	7	9	3
$H_6$	Mean	7.431E−03	**1.442E−13**	8.382E−04	5.693E−05	5.434E−03	4.852E+01	4.075E+01	4.133E+01	4.424E−01
	Std	(2.352E−02)	(9.112E−14)	(1.013E−03)	(3.712E−04)	(3.302E−03)	4.853E+01	(8.144E+00)	(6.325E+00)	(3.413E−01)
	rank	+	+	+	+	+	−	−	−	
	f-rank	2	4	3	1	5	8	7	9	6
$H_7$	Mean	1.801E+02	1.011E+02	8.094E+01	**6.323E+01**	1.233E+02	2.744E+02	2.223E+02	5.513E+02	1.430E+02
	Std	(1.972E+01)	(6.482E+00)	(3.783E+00)	(1.702E+00)	(1.274E+01)	(7.292E+01)	(3.495E+01)	(4.084E+01)	(2.024E+01)
	rank	−	+	+	+	+	−	−	−	
	f-rank	4	5	2	1	6	8	7	9	3
$H_8$	Mean	9.422E+01	5.524E+01	3.232E+01	**1.192E+01**	7.594E+01	3.294E+02	2.594E+02	4.825E+02	2.334E+02
	Std	(1.773E+01)	(7.763E+00)	(3.824E+00)	(2.272E+00)	(1.612E+01)	(7.293E+01)	(4.515E+01)	(4.673E+01)	(3.054E+01)
	rank	+	+	+	+	+	−	−	−	
	f-rank	5	3	2	1	4	8	7	9	6
$H_9$	Mean	4.832E+01	1.174E+00	1.112E+00	**0.000E+00**	7.384E+00	1.000E+04	1.062E+04	3.533E+04	9.325E+03
	Std	(6.293E+01)	(1.312E+00)	(9.371E−01)	(0.000E+00)	(5.773E+00)	(2.905E+03)	(3.103E+03)	(4.823E+03)	(1.254E+03)
	rank	+	+	+	+	+	−	−	−	
	f-rank	4	3	2	1	6	8	7	9	5
$H_{10}$	Mean	6.602E+03	3.754E+03	3.344E+03	**3.172E+03**	3.494E+03	7.103E+03	6.094E+03	7.394E+03	4.202E+03
	Std	(1.633E+03)	(2.542E+02)	(2.943E+02)	(2.543E+02)	(4.313E+02)	(5.343E+02)	(3.553E+02)	(3.261E+02)	(6.122E+02)
	rank	−	+	+	+	+	−	−	−	
	f-rank	6	3	2	1	5	8	7	9	4
$H_{11}$	Mean	1.092E+02	1.364E+02	1.202E+02	6.863E+01	6.744E+01	1.665E+02	1.183E+02	3.454E+02	**6.582E+01**
	Std	(3.542E+01)	(3.394E+01)	(2.933E+01)	(7.913E+00)	(8.724E+00)	(3.385E+01)	(1.915E+01)	(4.163E+01)	(1.063E+01)
	rank	−	−	−	−	−	−	−	−	
	f-rank	4	6	5	3	2	8	7	9	1
$H_{12}$	Mean	1.112E+05	5.143E+03	5.131E+03	2.163E+03	**1.293E+03**	1.000E+10	1.000E+10	1.000E+10	1.000E+10
	Std	(6.202E+04)	(3.324E+03)	(2.874E+03)	(4.512E+02)	(2.792E+02)	(0.000E+00)	(0.000E+00)	(0.000E+00)	(0.000E+00)
	rank	+	+	+	+	+	=	=	=	
	f-rank	5	4	3	2	1	6	6	6	6
$H_{13}$	Mean	1.212E+03	3.033E+02	2.652E+02	6.622E+01	**4.373E+01**	1.000E+10	9.803E+09	1.00E+10	9.213E+09
	Std	(1.451E+03)	(2.694E+02)	(1.494E+02)	(2.833E+01)	(1.762E+01)	(0.000E+00)	(1.404E+09)	(0.000E+00)	(2.793E+09)
	rank	+	+	+	+	+	−	−	−	
	f-rank	5	4	3	2	1	7	7	7	6
$H_{14}$	Mean	2.884E+03	1.054E+04	2.152E+02	2.764E+03	4.852E+01	2.051E+02	**3.984E+01**	3.264E+05	2.514E+03
	Std	(2.202E+03)	(3.112E+04)	(7.293E+01)	(4.511E+02)	(1.212E+01)	(2.135E+01)	(1.623E+01)	(1.603E+05)	(1.583E+02)
	rank	−	−	−	−	+	−	−	−	
	f-rank	7	8	5	6	2	4	1	9	3
$H_{15}$	Mean	3.352E+03	3.493E+02	3.224E+02	4.073E+01	4.464E+01	1.372E+09	2.853E+02	7.853E+09	**2.402E+01**
	Std	(2.793E+03)	(4.424E+02)	(1.423E+02)	(9.912E+00)	(1.123E+01)	(3.474E+09)	(3.544E+02)	(4.123E+09)	(5.992E+03)
	rank	−	−	−	−	−	−	−	−	
	f-rank	7	4	5	2	3	8	6	9	1

		SaDE^35^	JADE^35^	SHADE^52^	LSHADE^54^	MVMO^35^	CV1.0^29^	$CV_{new}$^53^	CS	MHDE
$H_{16}$	Mean	8.172E+02	8.563E+02	7.333E+02	**6.262E+01**	8.402E+02	1.535E+03	1.443E+03	1.763E+03	7.421E+02
	Std	(2.343E+02)	(1.754E+02)	(1.884E+02)	(2.833E+01)	(1.933E+02)	(2.744E+02)	(2.104E+02)	(2.372E+02)	(1.344E+02)
	rank	−	−	−	−	−	−	−	−	
	f-rank	5	6	4	1	3	8	7	9	2
$H_{17}$	Mean	5.483E+02	6.001E+02	5.164E+02	5.542E+02	5.192E+02	1.252E+03	**1.133E+02**	1.183E+03	4.032E+02
	Std	(1.533E+02)	(1.212E+02)	(1.112E+02)	(7.454E+01)	(1.333E+02)	(1.855E+02)	(1.924E+02)	(1.783E+02)	(1.183E+02)
	rank	−	−	−	−	−	−	−	−	
	f-rank	5	7	4	6	3	8	1	9	2
$H_{18}$	Mean	3.243E+04	1.893E+02	1.893E+02	3.923E+01	4.173E+01	5.212E+02	** 1.512E+02**	1.433E+06	5.903E+05
	Std	(1.682E+04)	(1.254E+02)	(1.034E+02)	(1.102E+01)	(1.944E+01)	(1.193E+02)	(4.434E+01)	(5.893E+05)	(6.123E+04)
	rank	+	+	+	+	+	+	+	−	
	f-rank	7	6	5	3	4	2	1	9	8
$H_{19}$	Mean	1.131E+04	3.241E+02	1.593E+02	2.454E+01	**1.733E+01**	1.734E+02	5.573E+01	1.99E+08	1.194E+02
	Std	(1.683E+04)	(1.252E+03)	(5.684E+01)	(8.814E+00)	(5.134E+00)	(4.173E+02)	(1.103E+01)	(1.393E+09)	(1.131E+03)
	rank	−	−	−	+	+	−	+	−	
	f-rank	7	5	6	2	1	8	3	9	4
$H_{20}$	Mean	3.523E+02	4.383E+02	3.332E+02	** 1.733E+02**	3.494E+02	1.054E+03	2.813E+02	1.043E+03	3.143E+02
	Std	(1.501E+02)	(1.332E+02)	(1.203E+02)	(7.923E+01)	(1.473E+02)	(2.143E+02)	(1.652E+02)	(1.673E+02)	(7.463E+01)
	rank	−	−	−	+	−	−	−	−	
	f-rank	5	6	4	1	7	8	2	9	3
$H_{21}$	Mean	2.873E+02	2.592E+02	2.334E+02	2.622E+02	2.772E+02	5.414E+02	**1.184E+02**	6.554E+02	2.264E+02
	Std	(1.362E+01)	(9.633E+00)	(5.113E+00)	(1.942E+01)	(1.605E+01)	(6.273E+01)	(8.773E+01)	(7.933E+01)	(1.143E+02)
	rank	−	−	+	−	−	−	−	−	
	f-rank	3	7	4	5	6	8	2	9	1
$H_{22}$	Mean	2.923E+03	3.332E+03	3.174E+03	2.49E+03	3.263E+03	7.333E+03	5.772E+03	8.193E+03	**1.000E+02**
	Std	(3.242E+03)	(1.802E+03)	(1.552E+03)	1.604E+03	(1.714E+03)	(1.993E+03)	(3.642E+02)	(4.083E+02)	(6.103E−03)
	rank	−	−	−	−	−	−	−	−	
	f-rank	2	3	4	5	6	8	7	9	1
$H_{23}$	Mean	5.222E+02	4.793E+02	4.593E+02	4.304E+02	5.044E+02	7.743E+02	**1.873E+02**	9.142E+02	6.144E+02
	Std	(2.053E+01)	(1.172E+01)	(8.754E+00)	(5.072E+02)	(1.715E+03)	(8.063E+01)	(5.113E+01)	(4.592E+01)	(4.764E+01)
	rank	−	+	+	+	+	−	+	−	
	f-rank	7	6	5	4	3	8	1	9	2

		SaDE^35^	JADE^35^	SHADE^52^	LSHADE^54^	MVMO^35^	CV1.0^29^	$CV_{new}$^53^	CS	MHDE
$H_{24}$	Mean	5.893E+02	5.313E+02	5.313E+02	5.62E+02	5.833E+02	8.322E+02	**3.252E+02**	1.012E+03	5.333E+02
	Std	(1.862E+01)	(7.622E+00)	(7.455E+00)	(2.334E+02)	(1.693E+01)	(1.213E+01)	(8.954E+01)	(6.383E+01)	(2.634E+01)
	rank	−	=	=	−	−	−	+	−	
	f-rank	7	5	4	6	3	8	2	9	1
$H_{25}$	Mean	5.713E+02	5.193E+02	5.063E+02	4.85E+02	5.093E+02	5.434E+02	**4.702E+02**	5.334E+02	4.312E+02
	Std	(3.052E+01)	(3.483E+01)	(3.644E+01)	(1.634E+01)	(3.123E+01)	(1.512E+01)	(2.264E+01)	(1.665E+01)	**(8.654E+00)**
	rank	−	−	−	−	−	−	+	−	
	f-rank	3	5	7	6	4	8	2	9	1
$H_{26}$	Mean	2.523E+03	1.612E+03	1.412E+03	1.14E+03	1.932E+03	2.484E+03	1.163E+03	4.574E+03	**3.215E+02**
	Std	(3.373E+02)	(1.213E+02)	(9.783E+01)	(4.493E+01)	(2.865E+02)	(1.884E+03)	(1.565E+03)	(1.823E+03)	**(2.412E−04)**
	rank	−	−	−	−	−	−	−	−	
	f-rank	8	4	5	6	7	3	2	9	1
$H_{27}$	Mean	7.101E+02	5.501E+02	5.494E+02	5.33E+02	5.434E+02	7.384E+02	**4.533E+02**	8.173E+02	5.000E+02
	Std	(6.653E+01)	(2.343E+01)	(2.782E+01)	(1.913E+01)	(1.753E+01)	(8.212E+01)	(7.174E+01)	(5.684E+01)	**(2.802E−01)**
	rank	−	−	−	−	−	−	+	−	
	f-rank	3	6	5	7	8	4	2	9	1
$H_{28}$	Mean	4.993E+02	4.912E+02	4.794E+02	4.733E+02	4.64E+02	4.944E+02	4.583E+02	5.125E+02	**4.002E+02**
	Std	(1.532E+01)	(2.083E+01)	(2.413E+01)	(2.243E+01)	(1.505E+01)	(1.932E+01)	(2.334E−01)	(1.882E+01)	**(7.563E−04)**
	rank	−	−	−	−	−	−	−	−	
	f-rank	6	3	4	2	5	8	7	9	1
$H_{29}$	Mean	5.111E+02	4.773E+02	4.874E+02	**3.512E+02**	4.894E+02	1.694E+03	1.453E+03	1.575E+03	7.282E+02
	Std	(1.373E+02)	(8.062E+01)	(1.052E+02)	(1.043E+01)	(1.403E+01)	(2.292E+02)	(1.684E+02)	(1.794E+02)	(1.503E+02)
	rank	+	+	+	+	+	−	−	−	
	f-rank	5	2	3	1	6	8	7	9	4
$H_{30}$	Mean	8.074E+05	6.683E+05	6.821E+05	6.534E+05	5.81E+05	4.642E+06	6.024E+05	2.952E+09	**3.972E+04**
	Std	(8.333E+04)	(9.252E+04)	(8.514E+04)	(7.323E+04)	(1.023E+04)	(8.593E+06)	(2.994E+04)	(4.593E+09)	**(5.242E+03)**
	rank	−	−	−	−	−	−	−	−	
	f-rank	8	4	5	6	7	2	3	9	1
w/l/t		9/20/0	11/17/1	13/16/0	15/14/0	14/15/0	1/26/2	7/20/2	0/27/2	
	Average f-rank	4.821	4.653	3.962	3.173	4.241	7.203	4.513	9	3.101
	Overall f-rank	7	6	3	2	4	8	5	9	1

## Real-world applications I: engineering design problems

Here, the effectiveness of the MHDE algorithm is assessed across a range of real-world optimization problems with diverse constraints. To handle constraints, a variety of techniques including decoder functions, repair algorithms, feasibility preservation and penalty functions are employed, as outlined in^55^. In this study, the focus is to opt for penalty functions due to their simplicity of implementation and widespread adoption. A common method for constraint management through penalty functions is detailed through a specific implementation, as illustrated in the equation below.

26 $$\begin{aligned} Minimize \hspace{2pt} f(\textbf{x}) = f(\textbf{x}) \pm \left( \sum _{i=1}^p a_i G_i(\textbf{x})+ \sum _{j=1}^qb_jH_j(\textbf{x})\right) \end{aligned}$$

27 $$\begin{aligned} G_i(\textbf{x})= max(0, g_i(\textbf{x}))^n \end{aligned}$$

28 $$\begin{aligned} H_j(\textbf{x})= |h_j(\textbf{x})|^{\lambda } \end{aligned}$$

The equalities are described by $g_i(\textbf{x})$ and the inequalities by $h_j(\textbf{x})$. $p$ and $q$ count the number of equality and inequality constraints. Constants $a_i$ and $b_j$ are positive. $n$ and $\lambda$ are set as 1 or 2. Utilizing a penalty function results in an elevation of the objective function value when constraints are breached. This creates an incentive for the algorithm to steer clear of infeasible areas and prioritize the exploration of feasible regions within the search space.

For performance evaluation, four engineering design problems including, (1) pressure vessel design, (2) rolling element bearing design, (3) tension/compression spring design, and (4) cantilever beam design, are used. The MHDE algorithm is compared with respect to some of the well-known algorithms including, artificial rabbit optimization (ARO)^55^, taguchi search algorithm (TSA)^56^, multi-strategy chameleon algorithm (MCSA)^56^, hybrid particle swarm optimization (HPSO)^57^, equilibrium optimizer (EO)^21^, evolution strategies (ES)^58^, grasshopper optimization algorithm (GOA)^59^, ($\mu + \lambda$) evolutionary search (ES)^60^, harris hawk optimizer (HHO)^56^, cuckoo search (CS)^55^, GCAII^55^, ant colony optimization (ACO)^55^, co-evolutionary DE (CDE)^60^, bacterial foraging optimization algorithm (BFOA)^61^, symbiotic optimization search (SOS)^62^, passing vehicle search (PVS)^63^, meerkat optimization algorithm (MOA)^64^, red panda optimizer (RPO)^65^, mine blast algorithm (MBA)^66^, moth flame optimizer (MFO)^56^, thermal exchange optimization (TEO)^67^, GCAI^55^, co-evolutionary differential evolution (CDE)^60^, seagull optimization algorithm (SOA)^68^, co-evolutionary particle swarm optimization approach (CPSO)^57^, and dynamic opposition strategy taylor-based optimal neighbourhood strategy and crossover operator (DTCSMO)^69^.

### Pressure vessel design

**Figure 1:**
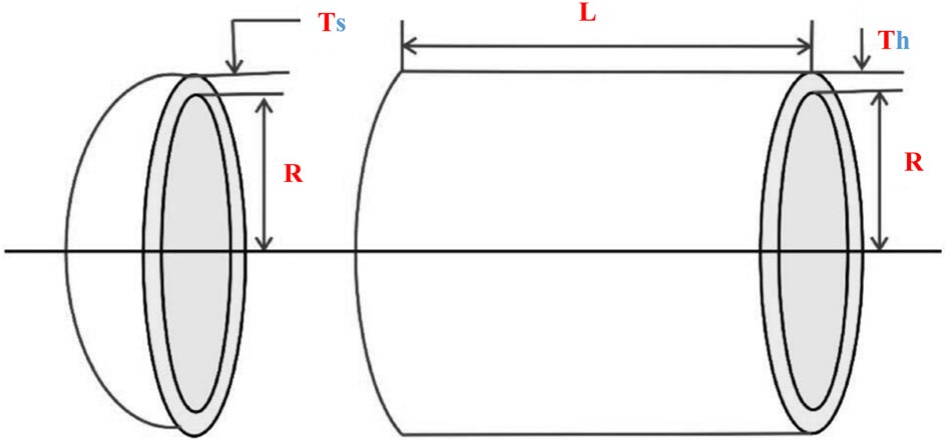
Pressure vessel design problem.

The optimization problem related to pressure vessels design is a widely acknowledged challenge in engineering. The fundamental objective is to minimize costs linked to material acquisition, welding, and the overall fabrication of pressure vessels, as discussed in^57^. This problem revolves around four key design variables: the thickness of the cylindrical shell represented as $T_s$, the inside radius of the cylindrical shell denoted as *R*, the head thickness of the cylindrical shell indicated by $T_h$, and the length of the cylindrical segment denoted as *L*. This problem has four constraints, as given by (29) and (30), as shown in Fig. 1.

Consider, $$\textbf{P}=\left[ L_1L_2L_3L_4\right] =\left[ T_sT_hRL\right] $$

29 $$\begin{aligned} Optimize, f\left( \textbf{P}\right) = 0.6224L_1L_2L_3+1.778L_2L_3^2+3.1661L_1^2L_4+19.84L_1^2L_3 \end{aligned}$$

30 $$\begin{aligned} \begin{aligned} Subjected \hspace{2pt} to, g_1\left( \textbf{P}\right)&=-L_1+0.0193L_3\le 0 \\ {P}_2\left( \textbf{P}\right)&=L_2+0.00954L_3\le 0 \\ L_3\left( \textbf{P}\right)&=\pi L_3^2L_4-\frac{4}{3}\pi L_3^2+1296000\le 0 \\ {P}_4\left( \textbf{P}\right)&=L_4-240\le 0 \end{aligned} \end{aligned}$$

Varying range, $$0\le L_1\le 99,\ 0\le L_2\le 99,\ 10\le L_3\le 200,\ 10\le L_4\le 200$$

**Table 6:** Statistical outcomes for Pressure vessel design challenge.

Algorithms	Optimal variables				Optimal cost
	$L_1$	$L_2$	$L_3$	$L_4$	
MHDE	0.7781695	0.3846499	40.32966	199.9994	**5885.3353**
CPSO^57^	0.8125	0.4375	42.091266	176.746500	6061.0777
HPSO^57^	0.8125	0.4375	42.098400	176.6366	6059.7143
ACO^70^	0.8125	0.4375	42.103624	176.572656	6059.0888
CDE^60^	0.8125	0.4375	42.098411	176.637690	6059.734
HHO^56^	0.810634392	0.395351718	41.43705230	185.0083559	6000.709575
MCSA^56^	0.778219825	0.384673362	40.32227070	199.9630859	5885.420268
LX-TLA^71^	0.7940	0.3930	41.1000	191.010	5960.010
EO^21^	0.8125	0.4375	42.0984456	176.6365958	6059.7143
HAIS-GA^72^	0.8125	0.4375	42.0931	176.7031	6060.367
HGA(2)^73^	1.1250	0.5625	58.1267	44.5941	6832.583
RPO^65^	0.778027	0.384579	40.31228	200	5882.8950
ES^58^	0.8125	0.4375	42.098087	176.640518	6059.745
BFOA^61^	0.8125	0.4375	42.096394	176.683231	6060.460
CDE^60^	0.8125	0.4375	42.098411	176.6377	6059.7340
TSA^56^	0.7783575016	0.385020966	40.32843029	199.8835841	5886.704101
MFO^56^	0.8125	0.4375	42.0984	176.6365	6059.7143
MVO^56^	0.789362835	0.392994906	40.94616932	191.5105526	5917.132761
MOA^64^	0.797811	0.390422	40.9048	192.016	5965.22494
ARO^55^	0.77824311	0.38475065	40.32338898	199.94794222	5885.667948
($\mu + \lambda$) ES^74^	0.8125	0.4375	42.098411	176.6366	6059.7340

**Figure 2:**
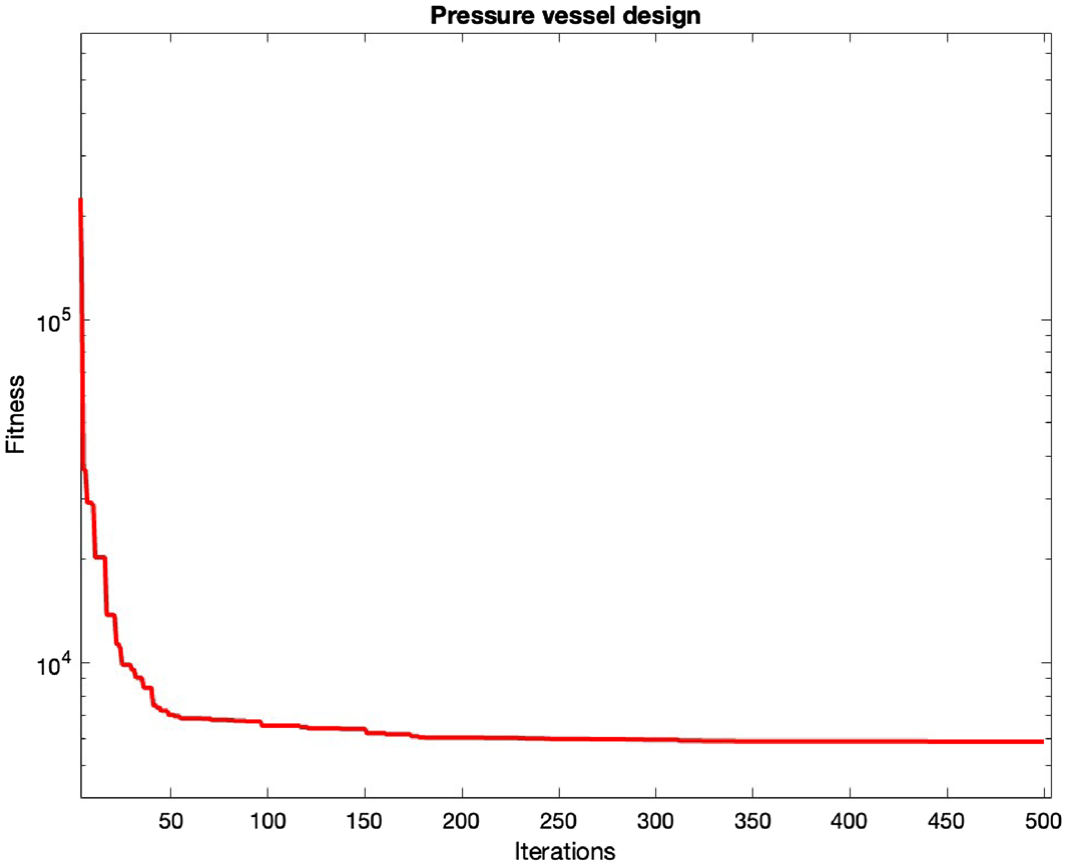
Convergence of pressure vessel design.

The outcomes pertaining to this design problem are presented in Table 6, where the results are evaluated using different algorithms for comparative analysis. These algorithms encompass MCSA^56^, ARO^55^, CPSO^75^, HPSO^57^, ($\mu + \lambda$)ES^60^, ACO^55^, CDE^60^, HHO^56^, MOA^64^, RPO^65^, MFO^56^, TSA^55^, MVO^56^, and others. The convergence patterns are shown in Fig. 2.

After optimization, the values of the variables obtained through MHDE are given by $x = (0.7781695, 0.3846499, 40.32966, 199.9994)$. The corresponding optimal cost for this design problem is $f = 5885.3353$. This result significantly outperforms the outcomes achieved by all other algorithms examined in our comparative analysis. The demonstration of such competitive performance serves to affirm the effectiveness of the proposed MHDE algorithm in comparison to the alternative algorithms that were evaluated.

### Rolling elemet bearing design

**Figure 3:**
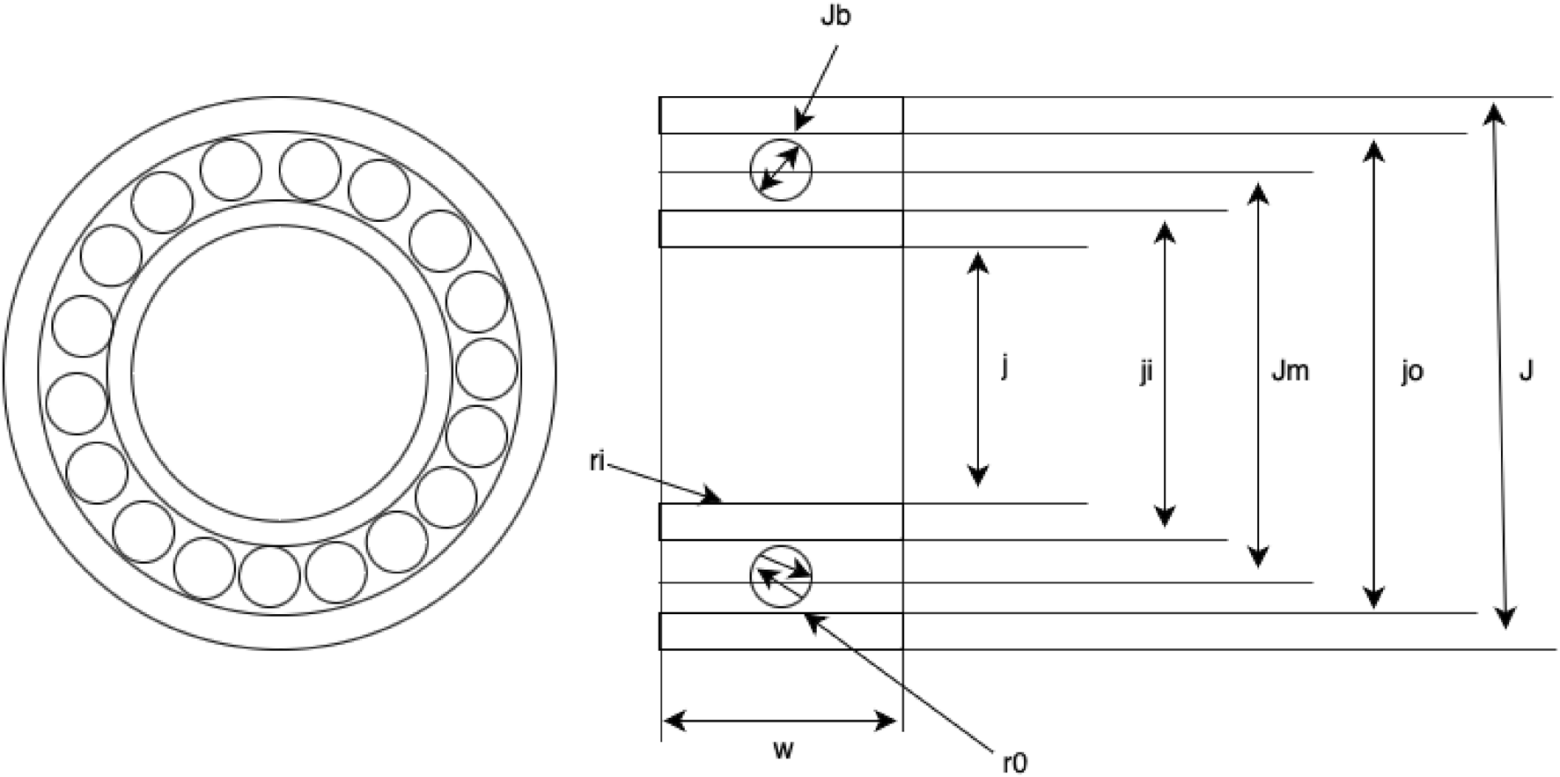
Rolling element bearing design challenges.

This optimization problem is associated with the load-bearing capacity of rolling elements^56^, and is represented in Fig. 3. This design problem has ten variables and many constraints. It is mathematically given by

Consider $$\textbf{P} = [J_m, J_b, Z, f_i, f_o, K_{Dmin}, K_{Dmax}, \epsilon , e, \zeta ]$$

31 $$\begin{aligned} Maximise \left\{ {\begin{array}{*{20}{l}} f_2(\textbf{P})= f_cP^{2/3}J_b^{1.8}&{} if\; J_b \le 25.4 mm\\ f_z(\textbf{P})= 3.647f_cP^{2/3}J_b^{1.4}&{} if\; J_b > 25.4 mm \\ \end{array}}\right. \end{aligned}$$

32 $$\begin{aligned} \begin{aligned} Subject \hspace{10pt} to \\ g_1(\textbf{P})&= \frac{\phi _0}{2sin^{-1}(J_b/J_m)}-P+ 1 \ge 0,\hspace{5pt} g_2(\textbf{P})= 2J_b- K_{Dmin}(J-d)\ge 0, \\ g_3(\textbf{P})&= K_{Dmax}(J-j)-2J_b \ge 0, \hspace{5pt} g_4(\textbf{P})= J_m-(0.5-e)(J+j)\ge 0, \\ g_5(\textbf{P})&= (0.5+e)(J+j)-J_m\ge 0, \hspace{5pt} g_6(\textbf{P})= J_m - 0.5(J+j)\ge 0, \\ g_7(\textbf{P})&= 0.5(J-J_m-J_b)-\epsilon J_b\ge 0, \hspace{5pt} g_8(\textbf{P}) = \zeta B_w -J_b \le 0, \\ g_9(\textbf{P})&= f_i \ge 0.515, \hspace{5pt} g_{10}(\textbf{P}) = f_o \ge 0.515 \end{aligned} \end{aligned}$$

where

33 $$\begin{aligned} f_c &= 37.91[1+\left\{ 1.04\left( \frac{1-\gamma }{1+\gamma }\right) ^{1.72}\left( \frac{f_i(2f_o-1)}{f_o(2f_i-1)}\right) ^{0.4}\right\} ^10/3]^{-0.3} \times \left( \frac{\gamma ^{0.3}(1-\gamma )^{1.39}}{f_o(1+\gamma ^{\frac{1}{3}})}\right) \left( \frac{2f_i}{2f_i-1}\right) ^{0.41} \end{aligned}$$

34 $$\begin{aligned} \gamma &= \frac{J_b}{J_m}, \hspace{5pt} f_i = \frac{r_i}{J_b}, \hspace{5pt} f_o = \frac{r_o}{J_b}, \end{aligned}$$

35 $$\begin{aligned} \phi _o &= 2\pi - 2cos^{-1} \frac{\left\{ (J-j)/2 -3(T/4)^2\right\} ^2+ \left\{ J/2-(T/4)-J_b \right\} ^2- \left\{ j/2+ (T/4)\right\} ^2}{2\left\{ (J-j)/2-3(T/4)\right\} \left\{ J/2-(T/4-J_b)\right\} } \end{aligned}$$

$$T = J-j-2J_b$$, $$J =160$$, $$j= 90$$, $$B_w = 30$$, $$r_i = r_o = 11.033$$

Variable range

$$0.5(J+j)\le J_m \le 0.6(J+j), 0.15(J-j)\le J_b \le 0.45(J-j), 4\le Z \le 50,$$

$$0.515\le f_i \le 0.6, 0.515\le f_o \le 0.6, 0.4 \le K_{Dmin}\le 0.5, 0.6 \le K_{Dmax} \le 0.7, 0.3 \le \epsilon \le 0.4,$$

$$0.02 \le e \le 0.1, 0.6 \zeta 0.85$$

**Table 7:** Statistical outcomes for Rolling element bearing design challenges.

Algorithms	Optimal variables										Optimal load carrying capacity
	$J_m$	$J_b$	Z	$f_i$	$f_o$	$K_{Dmin}$	$K_{Dmax}$	$\epsilon$	e	$\zeta$	
MHDE	125.7191	21.2716	11	0.5150	0.5150	0.4195017	0.6430438	0.3000	0.0310311	0.6963122	**85549.2391**
GA2^63^	125.7171	21.4231	11	0.5150	0.5150	0.4159	0.6510	0.3000	0.0223	0.7510	81843.3000
PVS^63^	125.7191	21.4256	11	0.5150	0.5150	0.4004	0.6802	0.3000	0.0800	0.7000	81859.74121
MBA^66^	125.7153	21.4233	11	0.5150	0.5150	0.4888	0.6278	0.3002	0.0946	0.6461	85535.9611
ARO^55^	125.7189	21	10.5403	0.5150	0.5150	0.4459	0.672132	0.3000	0.0825	0.6317	85548.5106
SOA^68^	125	21.4189	11	0.5150	0.5150	0.4000	0.7000	0.3000	0.0200	0.6000	85068.0520
DE	125.7192	21.2508	10.8654	0.5153	0.56021	0.4199	0.6197	0.3012	0.0472	0.6740	83629.26366
PSO	125.7251	21	11.2924	0.5153	0.5816	0.4569	0.6540	0.3092	0.0256	0.6128	81534.2759
TLBO^76^	125.7191	21.4256	11	0.5150	0.5150	0.4242	0.6339	0.3000	0.0689	0.7995	81859.7400
DTCSMO^69^	130.7576	18.0019	5.0265	0.6000	0.5954	0.4475	0.6458	0.3110	0.0554	0.6001	17021.1528

In this design example, the algorithms used for comaprion are ARO^55^, GA2^63^, MBA^66^, PVS^63^, TLBO^76^, SOA^68^, DTCSMO, PSO, and DE, and results are given in Table 7. The design variables obtained by MHDE for this particular scenario are determined as $$ x = (125.7191, 21.2716, 11, 0.5150, 0.5150, 0.4195017, 0.6430438, 0.3000, 0.0310311, 0.6963122)$$, and optimized cost is given as $$f = 85549.2391$$. From the results in Table 7, it can be seen that the proposed algorithm is highly competitive with respect to other algorithms.

### Tension/compression spring design

**Figure 4:**
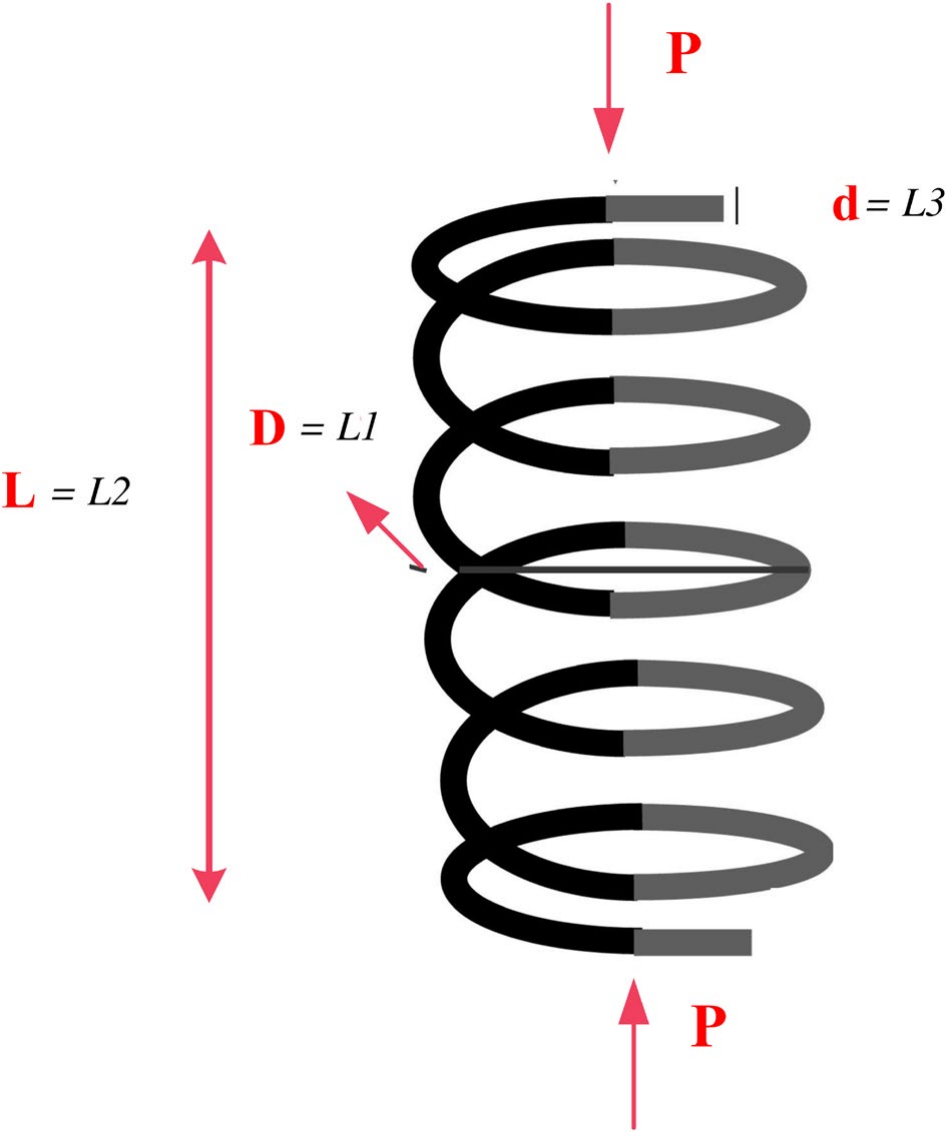
Tension compression spring design challenge.

For a compression spring, there are three design variables, including the wire diameter (*d*), mean coil diameter (*D*), and the number of active coils (*N*). The design is given in Fig. 4. The mathematical formulation is as:

Consider, $$\textbf{P}=\left[ L_1L_2L_3\right] =\left[ dDN\right] $$.

36 $$\begin{aligned} Minimize, \ f\left( \textbf{P}\right) =\left( L_1+2\right) L_2L_1^2 \end{aligned}$$

37 $$\begin{aligned} \begin{aligned}{}&Subjected \hspace{2pt} to, g_1\left( \textbf{P}\right) =1-\frac{L_2^3L_3}{71875L_1^4}\le 0 \\&\quad {g}_2\left( \textbf{P}\right) =\frac{4L_2^2-L_1L_2}{12566\left( L_2L_1^3-L_1^4\right) }+\frac{1}{5108L_1^2}\le 0 \\&\quad {g}_3\left( \textbf{P}\right) =1-\frac{140.45L_1}{L_3L_2^2}\le 0 \\&\quad {g}_4\left( \textbf{P}\right) =\frac{L_1+L_2}{1.5}-1\le 0 \end{aligned} \end{aligned}$$

Limits, $$0.005\le L_1\le 2.0,\ 0.25\le L_2\le 1.30,\ 2.0\le L_3\le 15.0$$

**Table 8:** Statistical outcomes for compression spring design challenges.

Algorithms	Optimal variables			Optimal cost
	$L_1$	$L_2$	$L_3$	
MHDE	0.0526768	0.380935	10	** 0.012684**
SCA^56^	0.051953681	0.363127167	11.92432679	0.012667794
TSA^55^	0.051849883	0.360542299	11.06962524	0.012668486
HPSO^57^	0.051706	0.357126	11.265083	0.0126652
LX-TLA^71^	0.0550	0.3600	1.0310	0.011
CPSO^57^	0.051728	0.357644	11.244543	0.012674
MFO^55^	0.05180485	0.359563992	11.12150367	0.012663719
SI^77^	0.050417	0.321532	13.97991	0.013060
BFOA^61^	0.051825	0.359935	11.107103	0.012671
CSA^56^	0.051552422	0.35349137	11.47804737	0.012663785
EO^21^	0.0516199100	0.355054381	11.38796759	0.012666
ARO^55^	0.05189732	0.36174867	11	0.01266602
RPO^65^	0.051689	0.356718	11.28897	0.012602
CDE^60^	0.051609	0.354714	11.410831	0.0126702
GWO^19^	0.051210248	0.345337024	11.98548282	0.012669398
HGA^78^	0.051302	0.347475	11.852177	0.012668
HHO^56^	0.053405295	0.342869942	12.14673591	0.012669676
MCSA^56^	0.05174419	0.358099507	11.20587013	0.012663522
($\mu + \lambda$)ES^74^	0.052836	0.384942	9.807729	0.012689
CDE^60^	0.051609	0.354714	11.410831	0.012670

**Figure 5:**
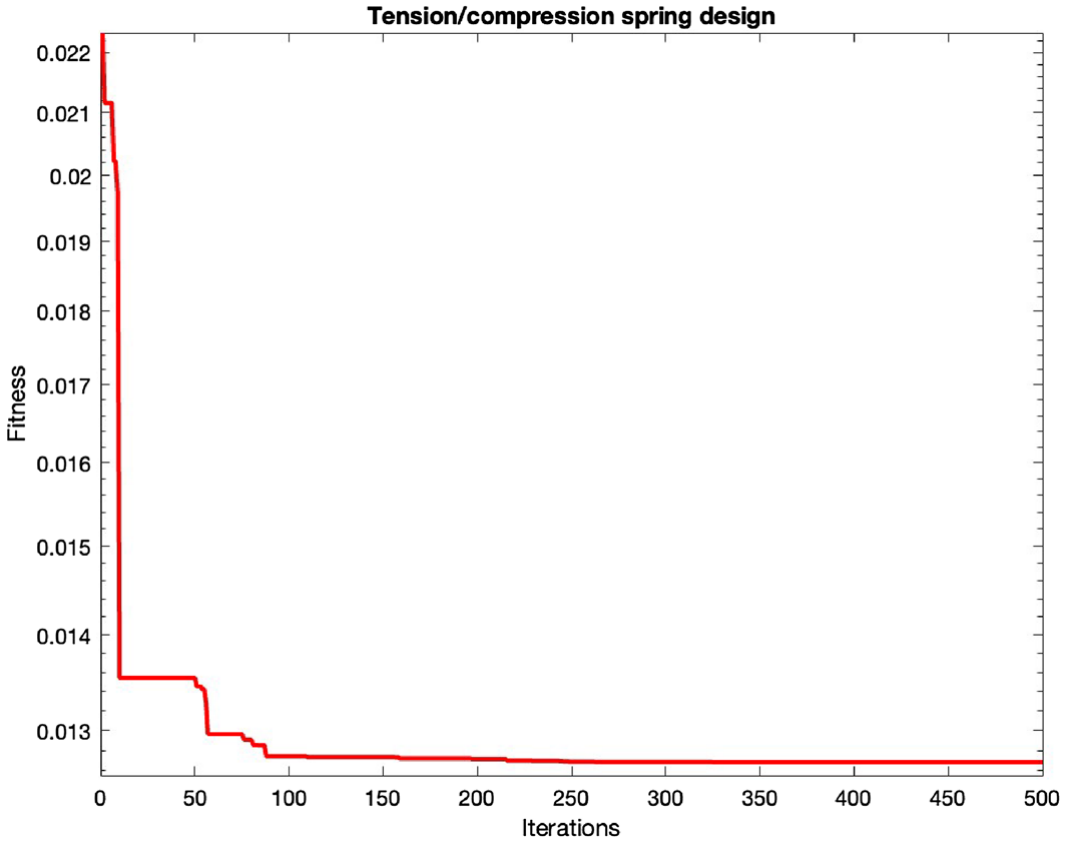
Convergence of tension/compression spring design.

In this scenario, a comprehensive comparison is conducted, with respect to RPO, CDE, GCAII^55^, MMA^79^, CPSO, SI, ARO^55^, SOS^62^, CS^55^, MFO^56^, GCAI^55^, MFO, BFOA, HHO, GOA^59^, and others, as outlined in Table 9. For this particular case, the optimal design variables derived using the MHDE algorithm are given in Table 8 and Fig. 5, are specified as $x = (0.0526768, 0.380935, 10)$. The resulting optimized cost is calculated as $f = 0.012684$. These results prove the significance of the proposed algorithm for tension spring design problem.

### Cantilever beam design

**Figure 6:**
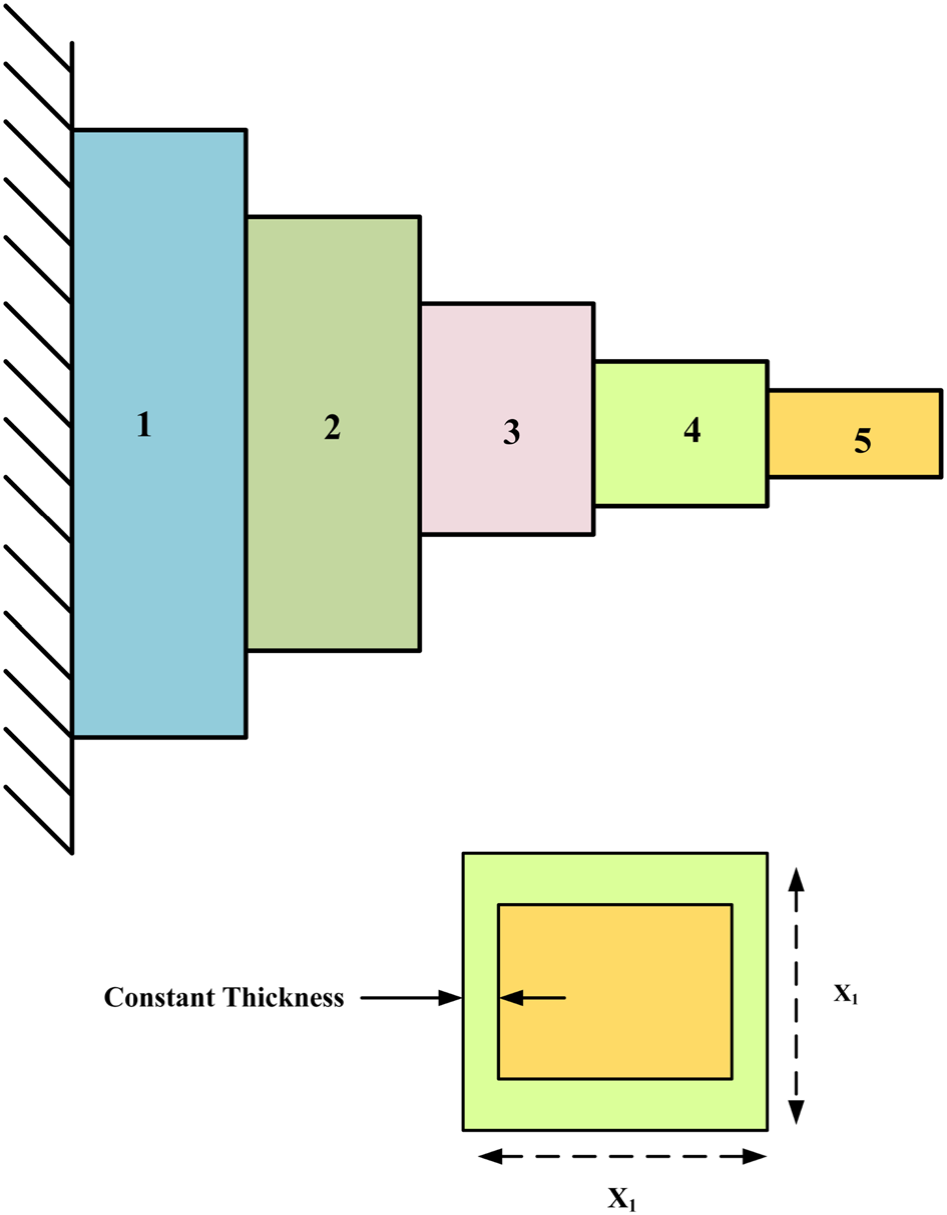
Cantilever beam design challenge.

This problem is meant for reducing the weight of a cantilever beam, having one constraint and five distinct blocks, representing several design variables, and is given by Fig. 6.

The design problem is mathematically given by Consider variable $\textbf{P}= [L_1, L_2, L_3, L_4, L_5]$

Minimize $$f_4(\textbf{P}= 0/0624(L_1+L_2+L_3+L_4+L_5))$$

Subject to $$g_1(\textbf{T})= \frac{61}{L_1^3}+\frac{37}{L_2^3}+\frac{19}{L_3^3}+\frac{7}{L_4^3}+\frac{1}{L_5^3}-1 \le 0$$

Variable range $$0.01 \le P_i \le 100, \hspace{5pt} i =1, \ldots , 5.$$

**Table 9:** Statistical outcomes for cantilever beam design challenges.

Algorithms	Optimal variables					Optimal cost
	$L_1$	$L_2$	$L_3$	$L_4$	$L_5$	
MHDE	6.0140	5.3128	4.4914	3.4993	2.1563	1.34000
MFO^22^	5.984871	5.316726	4.497332	3.513616	2.16162	1.339988
GCAI^55^	6.01000	5.30000	4.49000	3.49000	2.15000	1.34000
SOS^62^	6.01878	5.30344	4.49587	3.49896	2.15564	1.33996
MMA^79^	6.01000	5.30000	4.49000	3.49000	2.150000	1.340000
GCAII^55^	6.01000	5.30000	4.49000	3.49000	2.15000	1.34000
CS^55^	6.00890	5.30490	4.50230	3.50770	2.15040	1.39999
ARO^55^	6.00682	5.31143	4.493524	3.50289	2.15904	1.33996
GOA^59^	5.984871	5.31297	4.48307	3.50279	2.16333	**1.33996**

**Figure 7:**
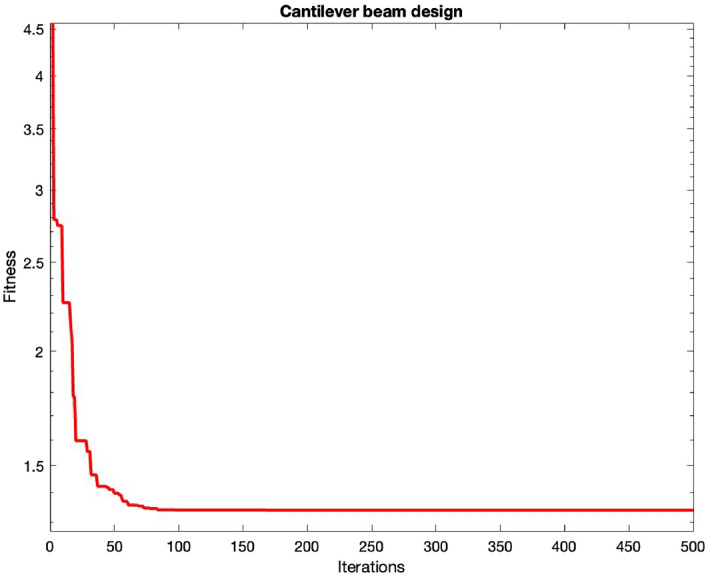
Convergence of cantilever beam design.

A comparison is performed with respect to MFO^56^, ARO^55^, SOS^62^, CS^55^, GCAII^55^, GCAI^55^, MMA^79^, and GOA^59^. The results, in Table 9, show that design variables for this problem are $x = (6.0140, 5.3128, 4.4914, 3.4993, 2.1563)$ and the optimized cost is $f = 1.34000$. The convergence patterns are given in Fig. 7. Here also, the proposed algorithm is significant with respect to others.

## Real-world applications II: frame design problems

Here, MHDE algorithm is used for weight minimization of 1-bay 8-story, 3-bay 15-story and 3-bay 24-story structures, respectively. The optimization results are compared with recently introduced hybrid algorithms to prove the significance of MHDE. Also, the frame structure benchmark problems are highly challenging to design due to higher level of difficulty in their implementation^80^. The figures of the three frames are taken from^81^.

Meta-heuristic algorithms (MHAs) have emerged as the core of modern optimization research and have set the trend for its use in almost every research domain. MHAs have been found to provide good solutions for frame design problems, and various algorithms have been presented in literature for optimal frame structure designing^28,36,82^. In the present work, the proposed MHDE is tested for optimizing weights in the frame structures. The termination criteria are based on maximum function evaluation and is inspired from^36^. The objective function is analysed 20,000 times for 1-bay 8-story frame and 30,000 for 3-bay 15-story frame whereas for 3-bay 24-story frame it is 50,000 respectively. For each problem, the population size used is 50 and a total of 20 independent runs have been performed. Apart from that, it has been kept in mind that there are no violations for a fair comparison among the algorithms. Here, a randomly generated initial population containing both feasible and infeasible solutions has been used to obtain statistically significant results.

### Designing 1-bay 8-story frame

**Figure 8:**
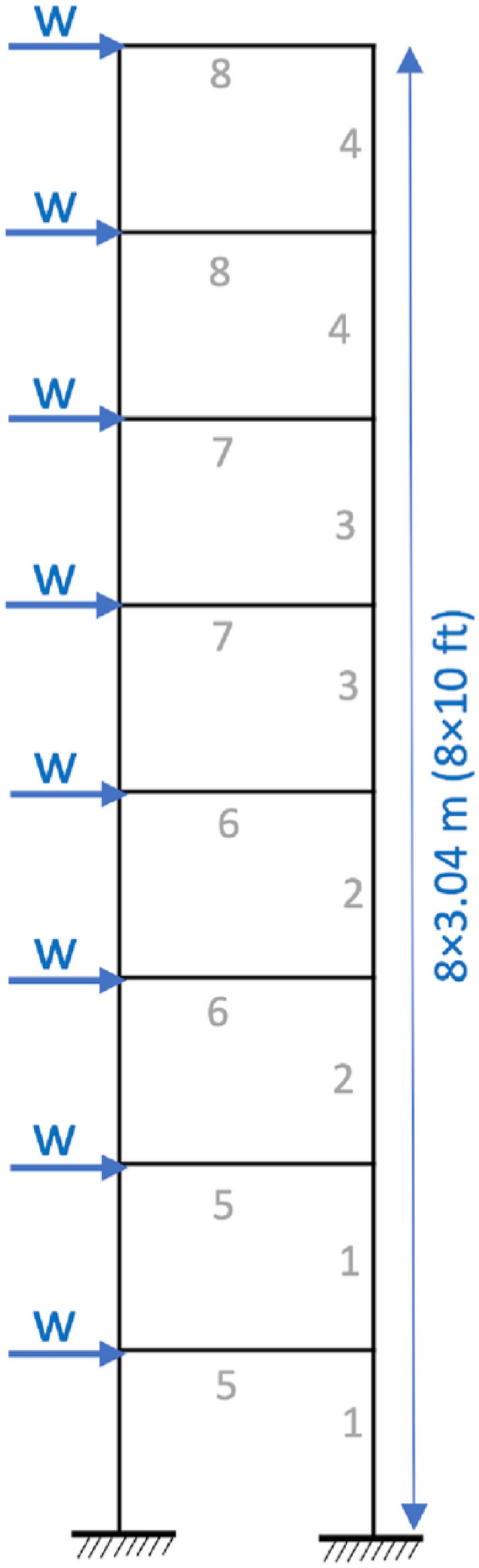
Design of 1-bay 8-story frame^81^.

For this case, fabrication conditions from the initial foundation steps are achieved by using the same beam section and same column section for every two successive stories. The modulus of elasticity for the material is E = 200 GPa (29000 ksi) and 267 W-shaped sections must be used for choosing cross-sectional areas of all the elements. The only constraint is that the latent drift must be less than 5.08 cm. The design is shown in Fig. 8.

**Table 10:** Optimization results for the 1-bay 8-story frame.

Element group	Optimal W-shaped sections								
	GA	DE	ES-DE	PSOACO	HGAPSO	PSOPC	SFLAIWO	ACO	MHDE
	^28^	^80^	^28^	^82^	^82^	^83^	^82^	^28^	
1	W18X35	W16X36	W18X40	W18X35	W18X35	W18X35	W18X40	W21X44	W14X38
2	W18X35	W16X36	W18X35	W16X32	W18X35	W14X26	W18X35	W18X35	W16X31
3	W18X35	W14X22	W14X22	W14X22	W14X22	W16X26	W14X22	W18X35	W8X28
4	W18X26	W12X22	W12X14	W12X16	W12X16	W14X16	W12X14	W12X22	W14X22
5	W18X46	W18X35	W18X46	W21X48	W16X31	W24X62	W18X35	W18X40	W10X33
6	W16X31	W16X31	W18X35	W18X40	W21X44	W18X35	W18X35	W16X26	W16X36
7	W16X26	W18X40	W18X35	W16X31	W18X35	W16X31	W18X35	W16X26	W16X31
8	W12X16	W14X30	W12X19	W16X36	W16X26	W12X30	W14X22	W12X14	W14X26
Weight (kN)	32.83	32.76	31.77	32.29	31.24	34.21	31.08	31.05	**30.70**

The comparison has been performed with respect to some of GA^28^, ACO^28^, DE^28^, ES-DE^28^, PSOACO^82^, HGAPSO^82^, PSOPC^82^ and SFLAIWO^82^. From the experimental results in Table 10, it has been found that MHDE has the minimum weight of 30.70 kN for the frame structure. The other best algorithms, ACO and SFLAIWO having 31.05 kN and 31.08 kN optimized weights respectively, are second and third best. Overall, MHDE provides more reliable results than most of the well-known algorithms reported in literature.

#### Designing 3-bay 15-story frame

**Figure 9:**
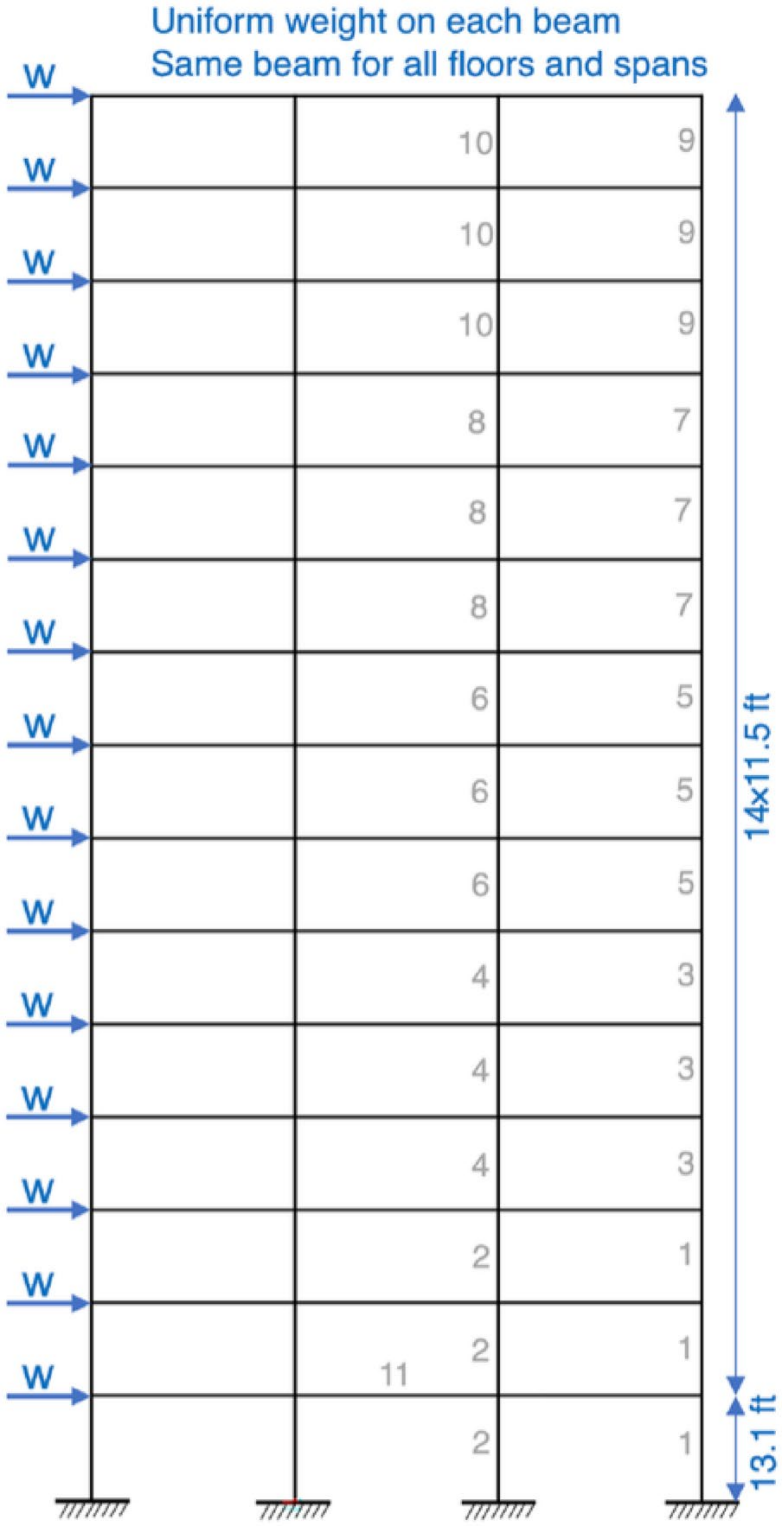
Design of 3-bay 15-story frame^81^.

For a 3-bay 15-story frame design, the ASIC combined strength constraint and displacement constraint is included as an optimization constraint. The material properties of the frame include: E = 200 GPa (29000 ksi), yield stress $F_y=248.2$ MPa and sway length on the top must be less than 23.5 cm. The length factor is calculated as $k_x \ge 0$ for sway permitted frame and the length factor out-of-plane is $k_y = 1.0$. The length of each beam is 1/5 span length and the design structure is given in Fig. 9.

**Table 11:** Optimization results for the 3-bay 15-story frame.

Element	Optimal W-shaped sections										
	HPSACO	ICA-ACO	HBB-BC	DE	AWEO	ES-DE	EVPS	SDE	SFLAIWO	FHO	MHDE
	^80^	^36^	^82^	^28^	^36^	^28^	^36^	^36^	^7^	^82^	
1	W21X111	W24X117	W24X117	W21X122	W18X143	W18X106	W14X99	W14X90	W14X90	W24X104	W10X54
2	W18X158	W21X147	W21X132	W33X141	W24X162	W36X150	W27X161	W36X170	W26X146	W33X152	W30X99
3	W10X88	W27X84	W12X95	W14X82	W24X84	W12X79	W24X84	W27X84	W18X76	W16X77	W14X53
4	W30X116	W27X114	W18X119	W30X108	W33X118	W27X114	W24X104	W24X104	W24X104	24X104	W24X68
5	W21X83	W14X74	W21X93	W30X108	W12X65	W30X90	W14X61	W14X61	W12X72	W14X74	W12X45
6	W24X103	W18X86	W18X97	W12X79	W18X97	W10X88	W30X90	W30X90	W18X86	W14X90	W16X57
7	W21X55	W12X96	W18X76	W14X61	W12X50	W18X71	W14X48	W14X48	W12X58	W14X61	W16X36
8	W27X114	W24X68	W18X65	W18X71	W21X68	W18X65	W12X65	W12X65	W14X61	W18X65	W12X40
9	W10X33	W10X39	W18X60	W6X25	W8X28	W8X28	W6X25	W6X25	W6X25	W6X20	W4X13
10	W18X46	W12X40	W10X39	W24X62	W16X40	W12X40	W12X40	W12X40	W16X36	W14X43	W10X26
11	W21X44	W21X44	W21X48	W21X48	W21X44	W21X48	W21X44	21X44	W21X44	21X44	W18X35
Weight(kN)	426.36	417.47	434.54	423.83	429.46	415.06	389.77	387.89	379.21	390.87	**360.22**

Here, nine improved algorithms are used for comparison including HPSACO^82^, HBB-BC^82^, ICA-ACO^36^, DE^28^, ES-DE^28^, AWEO^36^, EVPS^36^, FHO^7^, SDE^36^ and SFLAIWO^82^. From the optimization results in Table 11, it is evident that the minimum weight is obtained by MHDE and is equal to 360.22 kN. The second-best algorithm is SFLAIWO having an optimized weight of 379.21 kN whereas for the third best SDE it is 387.89 kN. In comparison to second best and third-best algorithm, MHDE has a reduced weight of 18.99 kN and 27.67 kN respectively. The optimized average of 20 runs for this frame using MHDE is 364.73 kN with a 2.16 kN std. This further proves the superiority of MHDE algorithm in comparison to others.

#### Designing 3-bay 24-story frame

**Figure 10:**
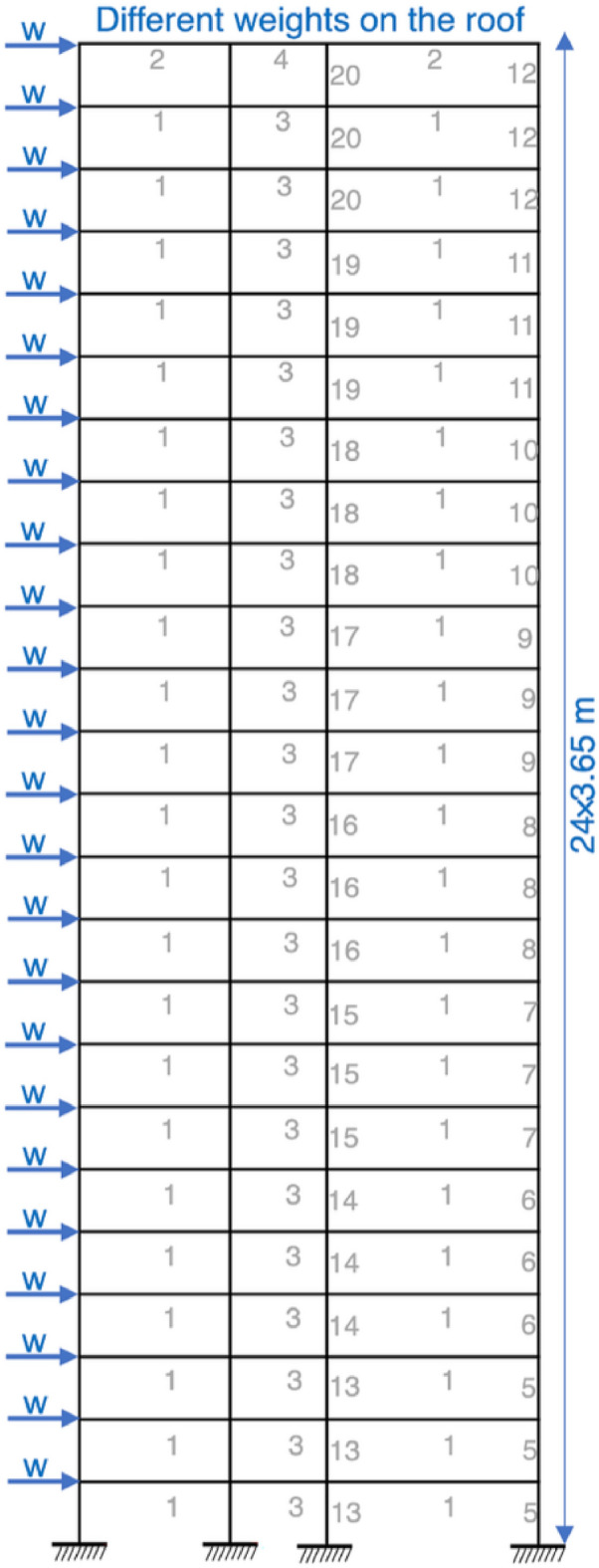
Design of 3-bay 24-story frame^81^.

This frame consists of 168 members^28^ and must be designed in accordance with LRFD specifications. This frame has a displacement constraint and properties of its material includes, $E=205$ GPa and $F_y=230.3$ MPa. The effective length is, $K_x \ge 0$ and the out-of-plane length is $K_y =1.0$. Here it should be noted that all the beams and columns are unbraced along the lengths and the design structure is shown in Fig. 10.

**Table 12:** Optimization results for the 3-bay 24-story frame.

Element	Optimal W-shaped sections										
	HS	HBB-BC	ICA	ICO	HBBPSO	ES-DE	AWEO	EVPS	SFLAIWO	FHO	MHDE
	^37^	^82^	^84^	^28^	^82^	^28^	^67^	^36^	^82^	^7^	
1	W30X90	W30X90	W30X90	W30X99	W30X90	W30X90	W30X90	W30X90	W30X90	W30X90	W30X90
2	W10X22	W21X18	W21X50	W16X26	W21X55	W21X55	W8X18	W6X15	W21X48	W21X48	W21X48
3	W18X40	W18X46	W24X55	W18X35	W21X48	W21X48	W24X55	W24X55	W21X48	21X48	W24X55
4	W12X16	W8X21	W8X28	W14X22	W27X24	W10X45	W26X8.5	W6X8.5	W21X48	W18X46	W14X43
5	W14X176	W14X176	W14X109	W14X145	W14X176	W14X145	W14X193	W14x159	W12X19	W14X159	W14X145
6	W14X176	W14X159	W14X159	W14X132	W14X90	W14X109	W14X120	W14X145	W14X176	W14X120	W14X132
7	W14X132	W14X109	W14X120	W14X120	W14X99	W14X99	W14X132	W14X90	W14X109	W14X109	W14X99
8	W14X109	W14X90	W14X90	W14X109	W14X99	W14X145	W14X82	W14X74	W14X109	W14X74	W14X90
9	W14X82	W14X82	W14X74	W14X48	W14X74	W14X109	W14X61	W14X74	W14X90	W14X68	W14X61
10	W14X74	W14X74	W14X68	W14X48	W14X74	W14X48	W14X38	W14X38	W14X48	W14X43	W14X48
11	W14X34	W14X38	W14X30	W14X34	W14X38	W14X38	W14X34	W14X30	W14X30	W14X30	W14X30
12	W14X22	W14X30	W14X38	W14X30	W14X34	W14X30	W14X22	W14X22	W14X34	W14X34	W14X38
13	W14X145	W14X159	W14X159	W14X159	W14X145	W14X99	W14X82	W14X99	W14X90	W14X99	W14X99
14	W14X132	W14X132	W14X132	W14X120	W14X132	W14X132	W14X109	W14X90	W14X120	W14X109	W14X99
15	W14X109	W14X109	W14X99	W14X109	W14X109	W14X109	W14X82	W14X99	W14X99	W14X99	W14X99
16	W14X82	W14X82	W14X82	W14X99	W14X90	W14X68	W14X82	W14X90	W14X90	W14X109	W14X82
17	W14X61	W14X68	W14X68	W14X82	W14X74	W14X68	W14X68	W14X68	W14X61	W14X74	W14X74
18	W14X48	W14X48	W14X48	W14X53	W14X48	W14X68	W14X68	W14X61	W14X53	W14X61	W14X53
19	W14X30	W14X34	W14X34	W14X38	W14X38	W14X61	W14X43	W14X43	W14X34	W14X38	W14X38
20	W14X22	W14X26	W14X22	W14X26	W14X22	W14X22	W14X34	W14X22	W14X22	W14X22	W14X22
Weight(kN)	956.13	960.90	946.25	967.33	941.55	945.15	927.59	905.67	911.78	910.72	**904.91**

For fabrication, the first and third bay of each floor uses the same beam section except the roof beam, and hence there are only 4 groups of beams. The initial stages of foundation, interior columns are grouped together over three consecutive stories. Overall, this frame consists of 4 groups of beams and 16 groups of columns, making the total number of design variables 20. The beam elements are chosen from 267 W-shapes, whereas column sections are restricted to W14 (37 W-shapes).

The optimized weights for this example are presented in Table 12. Here MHDE is compared with HBB-BC^82^, HS^37^, ICO^28^ ICA^84^, HBBPSO^82^, ES-DE^28^, AWEO^36^, FHA^7^, EVPS^36^ and SFLAIWO^82^. Here it has been found that among all the algorithms, MHDE achieved the minimum weight of 904.91 kN. The second and third best are EVPS and SFLAIWO algorithm and here the optimized weight is 905.67 kN and 911.78 kN respectively. The mean weight for 20 independent runs for MHDE is 910.23 kN with 3.78 kN deviation. The best values of results further prove the superiority of MHDE in contrast to other algorithms. Also, the function evaluations used for MHDE is much less than compared to other algorithms. For example, only, 50000 function evaluations are used for MHDE in contrast to SFLAIWO where 168,000 function evaluations have been utilized for weight optimization. Overall, it can be said that in this case also MHDE has superior performance and is easily able to enter the neighbourhood space of the global optimal solution.

## Conclusion

This article presented a multi-hybrid algorithm by combing the concepts of iterative division along with adaptive mutation for improved *expl*, adaptive parameter for a balanced *expl* and exploitation, population size reduction, and Gaussian random sampling for mitigating the local optima stagnation problem. The new optimization strategy helps to carry out global search in a more efficient way by using GWO based equations. All the above-discussed features ensure good performance of MHDE.

MHDE was evaluated using CEC 2005 classical benchmarks, CEC 2014 and CEC 2017 benchmark datasets. The experimental and statistical results prove that MHDE is superior with respect to DE variants such as JADE, SaDE, SHADE and others. The algorithm was then applied for weight minimization of three frames design problems with discrete variables. Optimization results prove the superior performance and competitiveness of MHDE over other algorithms for frame design also. To summarize, it is concluded that MHDE is reliable and an efficient algorithm for solving complex structural design problems.

Further studies should aim at providing theoretical analysis of the sensitivity and performance of MHDE. More work can be done to find a suitable combination of adaptive parameters to make the algorithm suitable for most of the domain research problems. Another possibility is to introduce some of the recent algorithms instead of GWO or CS for equation modifications, in order to control the search operation for better accuracy. Apart from that, combination of multiple strategies might lead to negative interference in algorithm’s behavior. For example, changing F factor will lead to different *expt*/*expl* performance, however if a good step size is achieved, it might not be carried out due to lack of a changed crossover permission. In this sense, a careful sensitivity analysis should be performed in order to verify interference of the proposed strategies combined. Finally, work on the convergence analysis can be performed to provide more insights into the working capabilities of the proposed algorithm.

## Data Availability

The datasets used and/or analysed during the current study available from the corresponding author on reasonable request.
